# Exercise intervention improves the modified Barthel index, Berg balance scale, and Fugl–Meyer assessment of upper limb motor function in stroke patients: a systematic review and meta-analysis

**DOI:** 10.1186/s13102-026-01764-z

**Published:** 2026-05-22

**Authors:** Zihan Zou, Yanjun Liu, Ziyi Zhang, Ran Zhao, Lei Gu

**Affiliations:** Hunan University of Chinese Medicine, No. 300 Xueshi Road, Yuelu District, Changsha, China

**Keywords:** Exercise Therapy, Stroke, Meta-analysis, Modified Barthel Index, Berg Balance Scale, Fugl–Meyer Assessment of Upper Limb Motor Function Scale

## Abstract

**Background and Objective:**

Exercise is a core approach in stroke rehabilitation, yet updated evidence on its effects on the Berg balance scale (BBS), modified Barthel index (MBI), and Fugl–Meyer assessment of upper extremity motor function (FMA-UE) remains limited. This study systematically evaluated the effects of exercise interventions on these three outcomes.

**Methods:**

PubMed, Web of Science, Cochrane Library, and China National Knowledge Infrastructure (CNKI) were searched from January 2015 to March 2025 for randomized controlled trials. Meta-analysis was performed using RevMan 5.4, and a meta-regression analysis was performed to explore the dose‒response relationship between exercise dose and improvements in FMA-UE scores. The minimal clinically important difference (MCID) was used to derive an optimal dose threshold.

**Results:**

Nineteen RCTs involving 1,041 participants were included. Exercise significantly improved the MBI (SMD = 0.95, 95% CI: 0.42–1.49), BBS (SMD = 0.73, 95% CI: 0.24–1.22), and FMA-UE (SMD = 0.62, 95% CI: 0.16–1.07) scores. The meta-regression analysis revealed a linear relationship (MD = 1.5252 + 0.0003 × total dose). The optimal dose threshold for achieving an MCID of 5 points was approximately 11,580 min (193 h).

**Conclusions:**

Exercise improves balance, daily activities, and upper limb function in stroke patients. The proposed “193-hour concept” provides a preliminary cumulative dose target for clinically meaningful upper limb recovery, pending prospective validation. PROSPERO registration: CRD420251272336.

**Supplementary Information:**

The online version contains supplementary material available at 10.1186/s13102-026-01764-z.

## Introduction

 Stroke is among the most common and disabling neurological disorders worldwide [1]. Epidemiological data consistently highlight its massive global disease burden; as stroke is a leading cause of long-term disability and mortality across all age groups, stroke incidence continues to increase in low- and middle-income countries [2]. Furthermore, the increasing prevalence of modifiable risk factors, such as hypertension, diabetes, and a sedentary lifestyle, further exacerbates its clinical impact [3]. This heavy disease burden not only severely decreases the quality of life of patients but also imposes immense socioeconomic pressure on global health care systems, underscoring the urgent need to identify effective rehabilitation strategies to improve the prognosis of patients with stroke [4]. In recent years, predictive modelling techniques represented by the RGX integrated model have been able to more accurately stratify the risk of death for stroke patients [5]. This progress further highlights the clinical urgency of developing and validating effective rehabilitation interventions to change the expected adverse outcomes.

Among the various poststroke rehabilitation interventions, exercise has emerged as a widely validated core approach [6] and is supported by robust evidence demonstrating its positive clinical effects. Regular and structured exercise not only enhances cardiovascular function and muscle strength [7], which are essential for mitigating the decline in physical function after a stroke, but also effectively promotes neuroplasticity, providing a neural foundation for the recovery of motor, sensory, and overall functions [8]. Compared with other adjunct therapies, such as manual therapy and electrical stimulation, exercise offers unique advantages. It is cost effective, easily implementable in both clinical and community settings, and empowers patients to transition from passive care recipients to active participants in their rehabilitation, thereby significantly improving the long-term sustainability of the intervention [9].

Standardized functional outcome measures are needed to provide a quantitative basis for an objective evaluation of the efficacy of exercise and other rehabilitation interventions. Currently, the modified Barthel index (MBI) [10], the Berg balance scale (BBS) [11], and the Fugl–Meyer assessment of upper limb motor function (FMA-UE) [12] are three of the most widely used core indicators in the field of stroke rehabilitation. Each scale offers unique and irreplaceable diagnostic value [13]. The MBI focuses on evaluating activities of daily living (e.g., eating, dressing, and walking) and serves as a fundamental tool for measuring the recovery of a patient’s independent living ability. The BBS specifically targets common poststroke balance dysfunctions, precisely quantifying a patient’s balance capacity and fall risk through 14 specific tasks. Moreover, the FMA-UE comprehensively assesses upper extremity motor domains, including fine motor skills, joint range of motion, and coordination, making it a critical metric for determining the extent of motor function recovery.

Given the central role of exercise in stroke rehabilitation and the importance of standardized metrics in evaluating therapeutic outcomes, this study aims to systematically review the effects of exercise interventions on MBI, BBS, and FMA-UE scores in stroke patients. Through this comprehensive meta-analysis, we intend to provide an updated evidence base to better guide clinical rehabilitation practice.

## Methods

### Research design and reporting standards

This review strictly adheres to the procedures stipulated in the Priority Reporting Items for Systematic Reviews and Meta-Analyses (PRISMA) guidelines [14]. The operational definitions were predefined in accordance with the recommendations of the Cochrane Manual on Systematic Reviews of Interventions [15]. The preset goals and methods have been registered on PROSPERO [CRD420251272336].

### Inclusion and exclusion criteria

The inclusion criteria were as follows: adult patients (aged ≥ 18 years) diagnosed by CT/MRI and meeting the diagnostic criteria for stroke from the World Health Organization, with the study type being a randomized controlled trial (RCT). Studies with repeated publications, incomplete data or an inability to extract valid information were excluded, and only those that assessed at least one of the MBI, BBS, or FMA-UE as the primary or secondary outcome measure were included.

The exclusion criteria were as follows: studies that were not RCTs (such as observational studies or reviews), studies whose results were repeatedly published, studies whose data could not be extracted, studies that were not published in Chinese or English, and studies that did not provide valid data (such as those that provided only charts without specific values).

### PICOS standard

The eligibility criteria were defined according to the PICOS framework and are summarized in Table 1.

**Table 1:** PICOS criteria for study inclusion

Element	Description
Population	Adult patients (aged ≥ 18 years) diagnosed with stroke by CT/MRI, regardless of the stroke stage (acute, subacute, or chronic). Patients with the following uncontrolled conditions that may confound the outcome assessment were excluded: acute neurological disorders such as brainstem or cerebellar lesions (which cause disproportionate ataxia or vestibular dysfunction severely interfering with MBI, BBS, and FMA-UE scoring) and Parkinson’s disease; cardiopulmonary diseases such as unstable angina and uncontrolled hypertension; uncontrolled metabolic diseases such as diabetes; and orthopaedic conditions such as severe joint contractures that preclude active exercise participation.
Intervention	Structured exercise intervention centred on physical activity, including aerobic training, resistance training, balance training, task-oriented training, as well as technology-assisted training (robot-assisted therapy and virtual reality training). Pure passive or cognitive interventions were excluded.
Comparison	Conventional rehabilitation care such as NDT, joint movement/muscle strength/daily session, including electrical stimulation and acupuncture, comforting activities such as watching videos without a therapeutic effect, matching intervention forms, health education, and stroke rehabilitation knowledge. The comparison group did not perform structured exercise or was not on a waiting list. If only basic medical care was maintained, a regular assessment was conducted, and no additional intervention was accepted.
Outcomes	The primary outcome measures were the modified Barthel Index (MBI), the Berg Balance Scale (BBS), and the Fugl–Meyer Assessment for the Upper Extremity motor function (FMA-UE).
Study design	Randomized controlled trials (RCTs). Observational studies, reviews, case reports, and nonrandomized designs were excluded.

The MBI is a standardized scale for assessing activities of daily living in stroke patients; it evaluates 10 core life skills, such as eating, washing, and dressing, and serves as a key indicator of rehabilitation efficacy and the prognosis [16]. The BBS assesses static and dynamic balance control and fall risk through 14 standardized actions, including sit–stand transitions and single-leg standing [17]. The FMA-UE evaluates upper limb motor recovery across 33 items, encompassing the joint range of motion, muscle strength, coordination, and reflexes [18].

### Retrieval strategy

We systematically searched the following electronic databases for publications from January 1, 2015, to March 31, 2025: PubMed, Web of Science, Cochrane Library, and China National Knowledge Infrastructure (CNKI). CNKI was used to avoid regional publication bias. Chinese-language studies were excluded after the full-text screen for the following reasons: (1) inadequate randomization (e.g., allocation by admission order); (2) interventions limited to acupuncture or herbal medicine without structured exercise; (3) only within-group comparisons reported, without between-group data; and (4) duplicate publications. The PubMed search strategy was as follows: (stroke OR cerebrovascular accident OR hemiplegia OR hemiparesis) AND (exercise OR exercise therapy OR physical activity OR aerobic training OR resistance training OR balance training OR task-oriented training OR robot-assisted OR virtual reality OR rehabilitation) AND (modified Barthel index OR MBI OR Berg Balance Scale OR BBS OR Fugl–Meyer assessment OR FMA-UE OR FMA) AND (randomized controlled trial OR RCT). Filters were applied for publication date (January 1, 2015, to March 31, 2025) and language (English or Chinese). Full search strategies for all four databases are presented in Supplementary Table S8. All strategies were peer-reviewed by a second reviewer prior to execution. A re-run of the searches immediately before finalising the review confirmed that no additional eligible studies were identified. A supplementary search was conducted by tracing the references of the included studies, and the grey literature was not searched for resource accessibility reasons. We clarify this limitation in the Discussion section.

### Study selection and data extraction

Two reviewers independently reviewed the initial list of studies and screened out duplicate and irrelevant studies based on the titles and abstracts. For studies where the full text could not be directly obtained, we attempted to acquire it through specific methods, such as contacting the corresponding author or using the library’s document delivery service. If it still could not be obtained, it was recorded as “Full text cannot be obtained” in the flowchart and excluded. Moreover, its potential impact was discussed in the Limitations section. Two reviewers conducted a rigorous analysis of the full texts of the remaining studies to determine the final included studies and extracted the data into a predesigned spreadsheet. A quantitative meta-analysis was conducted using the mean differences and SDs of the measured values of the three indicators of the MBI, BBS, and FMA-UE between the intervention group and the control group in RevMan 5.4 (see the Supplementary Materials). The extracted data included basic information on the study (author and publication year), characteristics of the research subjects (sample size, onset time, and training period), details of the intervention plan (intervention period, type of exercise, initiation time, duration, weekly frequency, and implementation location), control measures, and the MBI and BBS scores of the experimental group and the control group, respectively. Any disputes regarding the study selection and extraction of FMA-UE scores (means and standard deviations) were jointly discussed and resolved by the two reviewers and adjudicators. The classification was performed by a third reviewer.

### Assessment of the risk of bias

**Fig. 1:**
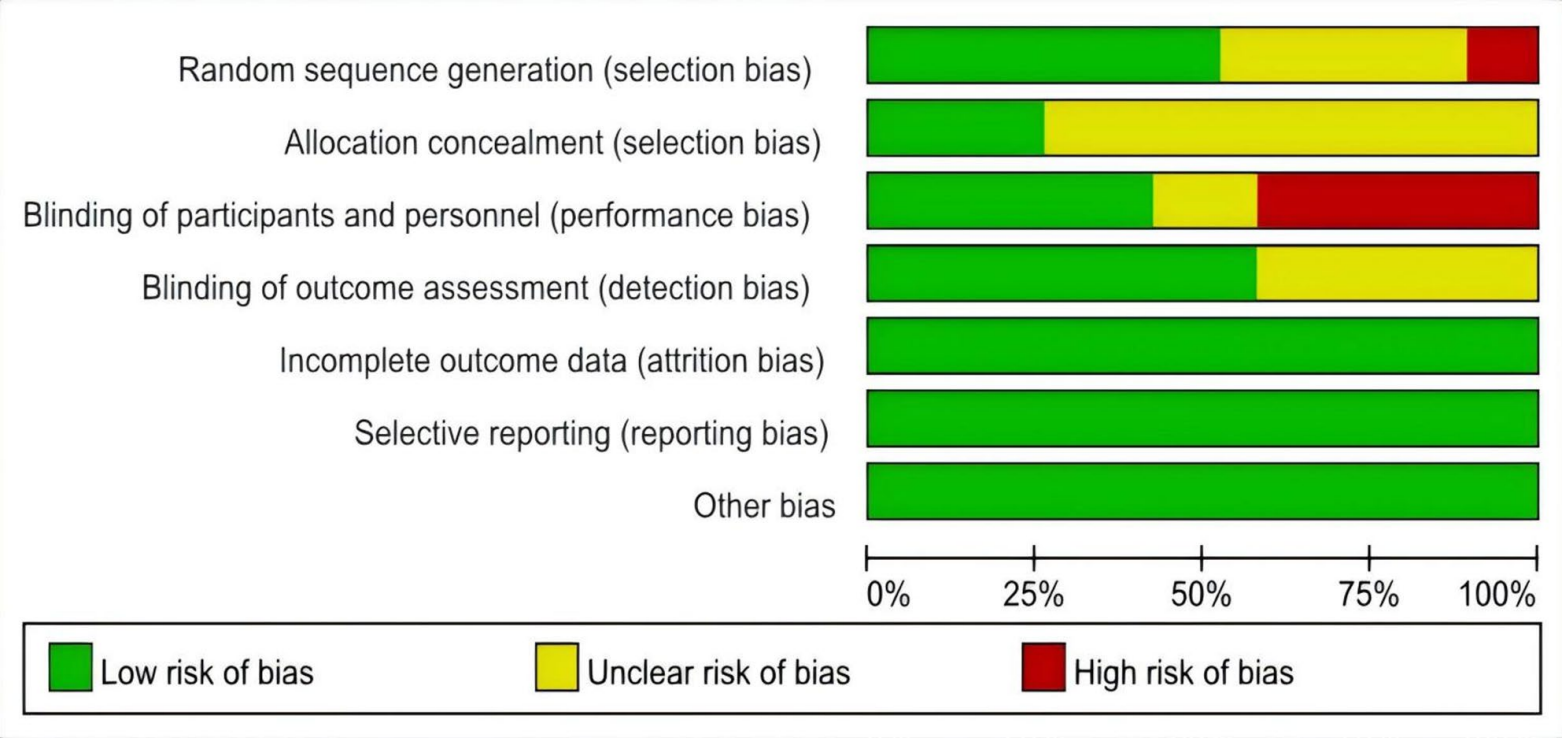
Chart summarizing the results of the risk of bias analysis

**Fig. 2:**
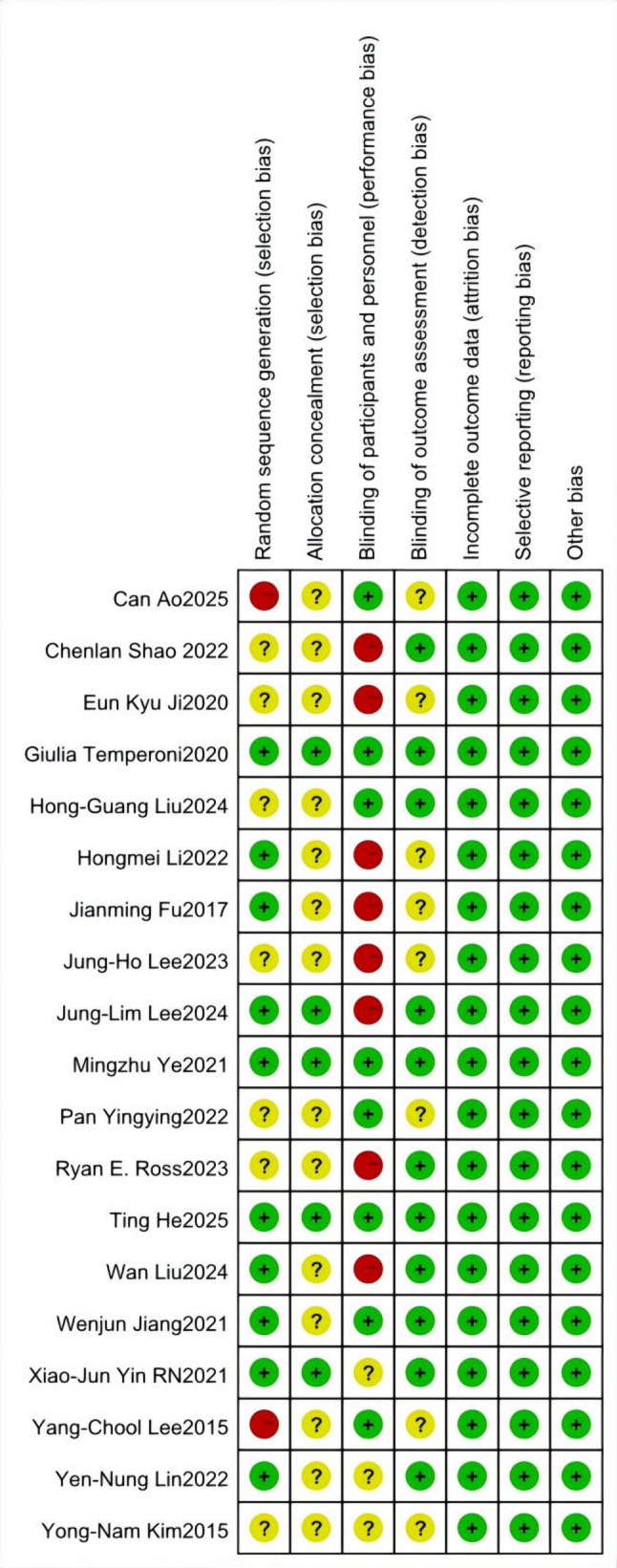
Risk of bias map

Two reviewers independently examined each study that met the inclusion criteria using the Risk of Bias assessment Tool (ROB2) checklist developed by the Cochrane Collaboration [19]. This tool assesses whether research bias can be attributed to the randomization process, deviated treatment groups, compliance, missing outcome data, outcome measurement, and outcome reporting. Research that is considered high risk in any of the above fields or has certain risks in multiple fields is classified as overall high-risk research. Research in any of the above fields that is considered to have certain risks is classified as medium-risk research. Studies that have low risks in all fields are classified as overall low-risk studies. This system describes the level of certainty of the research results based on the risk of bias, imprecision, inconsistency, inappropriateness, publication bias, effect size, dose responsiveness, and adjustments for confounding factors. Due to the nature of exercise interventions, most studies are at high risk of blinding of both participants and intervention personnel. If the outcome assessors were not blinded and the outcome indicators were functional scales with strong subjectivity, a risk of bias might be introduced in the field of outcome measurement. The risk of bias included in the study is shown in Figs. 1 and 2. Methodological quality was additionally assessed using the PEDro (Physiotherapy Evidence Database) scale, which is specifically designed for evaluating physical therapy trials. The PEDro scale consists of 11 items, of which Items 2–11 are scored from 0 = criterion not satisfied to 1 = criterion satisfied, yielding a total score ranging from 0 to 10. Item 1 (eligibility criteria specified) is not scored because it pertains to external validity. Two reviewers independently scored each study, and disagreements were resolved by a third reviewer. The certainty of evidence for each primary outcome was assessed using the Grading of Recommendations Assessment, Development and Evaluation (GRADE) framework. Starting from a baseline of “high certainty” for RCTs, the evidence was downgraded based on five criteria: risk of bias, inconsistency, indirectness, imprecision, and publication bias. A summary of the GRADE findings is presented in Supplementary Table S7.

### Statistical analysis

The research characteristics and results were synthesized using qualitative methods and summarized in table form. Using RevMan 5.4 software, a quantitative meta-analysis was conducted by calculating the standardized mean differences in the MBI, BBS, and FMA-UE scores between the intervention group and the control group. When the standardized mean difference was not provided in the published studies, we directly contacted the authors to obtain the raw data. If the original data still could not be obtained, then indirect methods were used to estimate these values. For each study, the data were extracted from the first postintervention assessment, which was defined as the measurement performed immediately after completion of the treatment period. Follow-up data collected after additional time intervals were not included in the primary analysis. The timing of this postintervention assessment varied across studies (e.g., at 4, 6, or 8 weeks), and this variation was examined as a potential source of heterogeneity through subgroup analyses stratified by the intervention period. If differences in units or scales were observed, the standardized mean difference (SMD) was calculated to combine the results, and the 95% confidence interval (CI) was calculated for both. For studies involving multiple effect sizes, the average value was calculated to represent the overall effect. If the study included multiple exercise treatments that varied in duration, the treatment group with the longest duration was compared with the control group. If multiple groups involving different exercise patterns, for example, aerobic exercise, resistance exercise, or a combination of both, the combined treatment group was compared with the control group in the primary analysis. We used the I² statistic to assess the heterogeneity among studies, and I² > 50% was considered to indicate significant heterogeneity [20]. Regardless of the level of heterogeneity, random-effects models are preferred for a pooled analysis because they are more conservative and can better accommodate variations among studies. When significant heterogeneity was observed, we explored its possible sources through predefined subgroup analyses (such as the intervention period and stroke stage). Sensitivity analyses were conducted to assess the robustness of the primary results. A leave-one-out analysis was performed for each outcome by iteratively removing one study at a time and recalculating the pooled effect estimate. Additionally, a quality-based sensitivity analysis was performed by excluding studies with a PEDro score less than 6. For subgroup analyses, the stroke stage (subacute vs. chronic) and type of exercise intervention (conventional, technology-assisted, and mind-body/cognitive-motor training) were explored as additional variables. All effect sizes for continuous outcomes in both the main and subgroup analyses were calculated as the SMDs to ensure consistency, given that minor differences in scale versions existed across studies.

### Interventions for upper limb movements and dose–effect analysis

#### Extraction of core intervention parameters and quantification of “total intervention dose”

Based on the conventional data extraction, this study further extracted the precise dose parameters of the exercise intervention analysed in the included literature in a structured manner, as shown in Table 2. Two researchers independently recorded the duration of a single exercise session (minutes), weekly intervention frequency (times/week), and total intervention period (weeks) of the experimental group. The “total intervention dose ” was defined as the core independent variable for the meta-regression analysis to comprehensively evaluate the cumulative effect of the exercise intervention. The formula for calculating the total intervention dose was as follows: total intervention dose (minutes) = single exercise duration × weekly intervention frequency × total intervention period.

**Table 2:** Data table

Study	Dose (minutes)	Mean Difference	Standard Error	Weight
Can Ao	12150	5.44	0.6285	2.53
Eun Kyu Ji	1680	0.44	5.9319	0.028
Hongmei Li	840	0.93	0.6787	2.17
Jung-Lim Lee	1200	4.55	1.7252	0.336
Mingzhu Ye	5760	4.92	2.1927	0.208
Ryan E. Ross	630	1.6	3.9147	0.065
Wenjun Jiang	600	3.06	1.2284	0.662

#### Meta-regression analysis and derivation of the MCID threshold

Considering the high heterogeneity (I^2^ = 80%) observed in the conventional meta-analysis of upper limb motor function (FMA-UE), a meta-regression analysis was performed in this study to explore the potential “dose‒response” relationship between the intervention dose and clinical efficacy.

Statistical model The nonstandardized mean difference (mean difference, MD) rather than the standardized mean difference (standardized mean difference, SMD) was used as the dependent variable, with the “total intervention dose (minutes)” as the continuous independent variable to retain the clinical interpretability of the original scale. The regression model was constructed using weighted least squares (WLS), and the weights for each independent study were assigned based on the inverse of the variance to ensure that studies with large samples and low variability had a greater contribution to the model.

Evaluation of the model The goodness of fit and explanatory power of the model were evaluated using the R^2^ statistic, and the significance of the predictor was defined as *p* < 0.05. The model was fitted using weighted least squares (WLS) with inverse-variance weights (1/SE²). The goodness of fit was evaluated with R², which was calculated as R² = 1 − SSE/SST, where SSE is the residual sum of squares and SST is the total sum of squares. The significance of the dose coefficient was assessed using a t test. A quadratic term (dose²) was added to test for potential nonlinear relationships. The nonsignificance of the quadratic term was interpreted as support for the linear model, although the limited sample size constrains the ability to detect subtle departures from linearity. Cook’s distance was determined to identify influential cases.

**Fig. 3:**
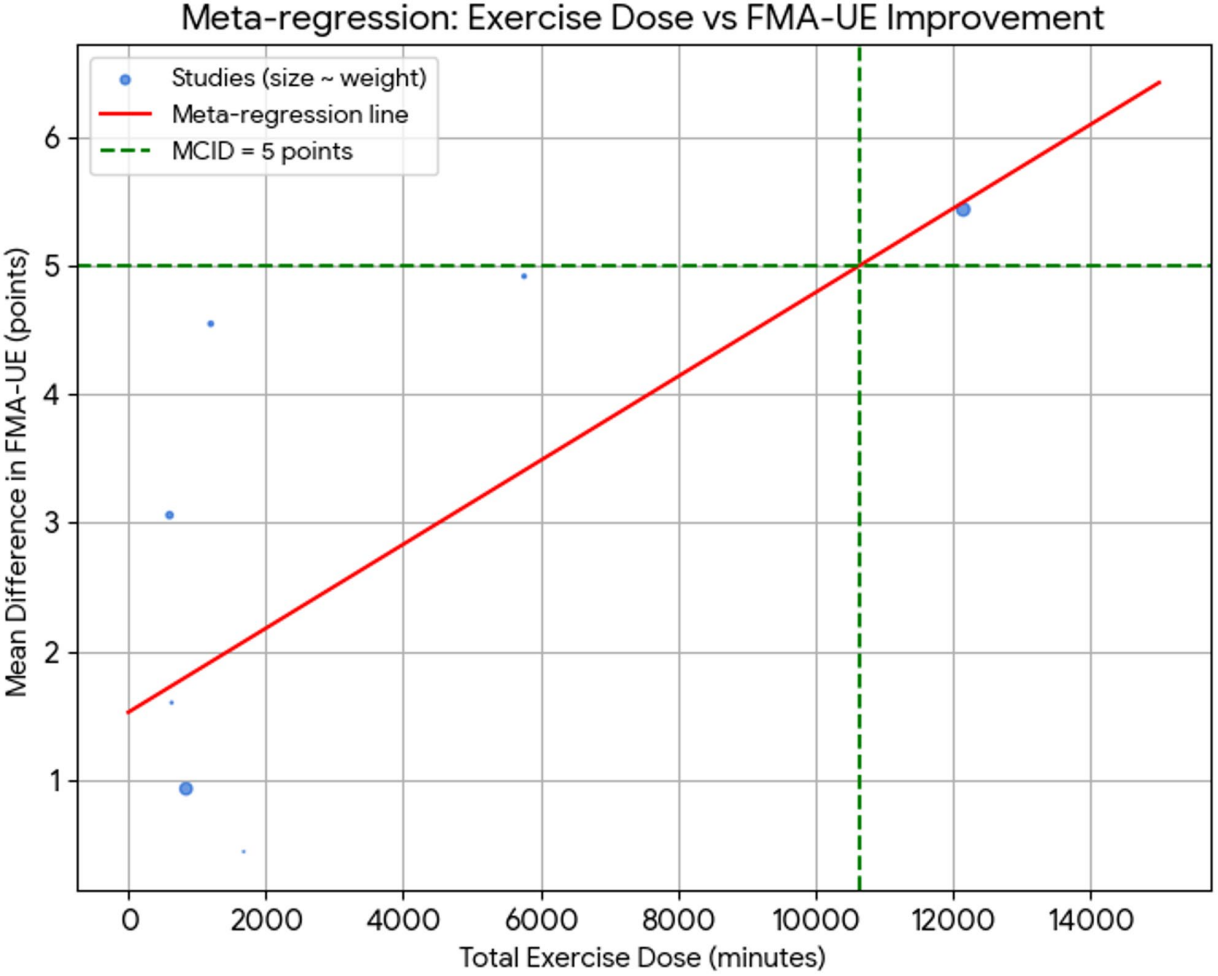
Scatter plot showing the results of the meta-regression analysis of the FMA-UE score

Derivation of the clinical threshold In this study, the concept of the minimum clinically important difference (MCID) was introduced to increase the clinical translatability of the research results. In accordance with the consensus in the field of stroke neurorehabilitation, the MCID of the FMA-UE score was set at 5 points. This threshold was substituted into the fitted regression equation (MD = β0 + β1 * Dose) shown in Fig. 3, and the “optimal total exercise dose threshold” that could result in a substantial clinical improvement in the upper limb function of stroke patients was calculated by reverse derivation.

## Results

### Literature screening process

**Fig. 4:**
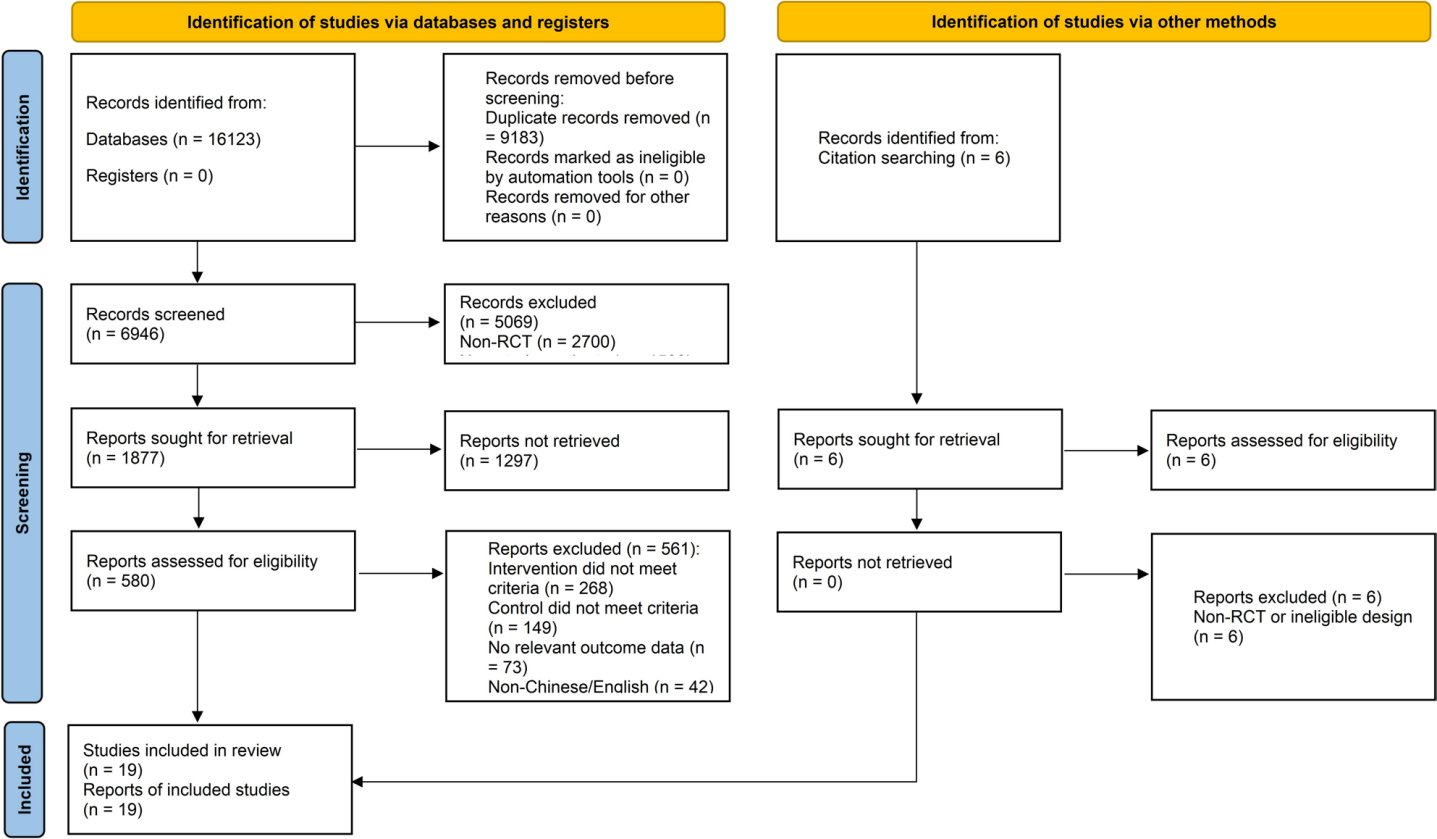
Flowchart of the literature screening process

This study strictly followed the PRISMA 2020 guidelines to screen the literature. Four databases, namely, PubMed, Web of Science, the Cochrane Library and China National Knowledge Infrastructure (CNKI), were systematically searched. The retrieval period was from January 1, 2015, to March 31, 2025. In addition, by manually tracing the list of references included in the study, six potentially relevant records were supplemented and retrieved. The complete search formula can be found in the Supplementary Materials. A total of 16,129 records were obtained during the initial inspection (16,123 were retrieved from the database, and 6 were from other sources). All records were imported into Medical Literature King management software. After automatic duplicate checking and manual verification and the elimination of duplicate records, the remaining 6,946 independent records were included in the screening process. First, two reviewers independently read the titles and summaries of these 6,946 records and conducted an initial screen based on the pre-established PICO standards. A total of 5,069 records were excluded at this stage. The main reasons for exclusion included the following: (1) nonrandomized controlled trials (such as reviews, meta-analyses, and observational studies; *n* = 2,700); (2) the research subjects were nonstroke patients (*n* = 1,500); (3) the intervention measures were not the exercise interventions defined in this study (*n* = 800); and (4) other obviously unrelated studies (*n* = 69). The remaining 1,877 records were considered potentially eligible and were entered into the full-text acquisition and evaluation stage. We have made every effort to obtain the full texts of the abovementioned 1,877 articles through database downloads and subscriptions to institutional libraries and by contacting the authors. Among these records, 1,297 articles were excluded because of unavailability (for example, only abstracts, invalid links, or an inability to obtain the articles through legal channels). Ultimately, a total of 580 documents were successfully obtained and included in the detailed full-text evaluation. Two reviewers conducted independent and rigorous evaluations of the full texts of these 580 articles. After a careful review, a total of 561 studies were excluded. The specific reasons for exclusion are as follows: the intervention measures did not meet the inclusion criteria (for example, pure passive therapy, cognitive training, or no structured physical activity components, *n* = 268); the control group did not meet the requirements (for example, the control performed another active exercise intervention instead of being an unconventional rehabilitation/consolation control, *n* = 149); no relevant outcome data or data could be extracted (available data for MBI, BBS or FMA-UE scores were not reported, *n* = 73); non-Chinese and non-English full texts (*n* = 42); and studies with repeated publication or overlapping data (*n* = 29). Ultimately, 19 randomized controlled trials fully met all the inclusion criteria and were included in this systematic review for qualitative synthesis. Since all 19 studies reported complete data available for the meta-analysis, they were entered into a quantitative synthesis (meta-analysis). The detailed screening process is shown in Fig. 4.

### Characteristics of the studies

A total of 19 randomized controlled trials involving 1,041 participants met our updated inclusion criteria. The MBI was used in 10 studies [21–30], 7 studies assessed the BBS [23, 26, 29–33], and the FMA-UE was used in 7 studies [29, 34–39]. Key characteristics of the included studies are summarized below.

- Population: The studies covered patients from the acute phase (within 6 months after stroke) to the chronic phase (over 6 months).
- Intervention types: The interventions fell into three main categories. Conventional approaches included bilateral upper limb training, task-oriented training combined with acupuncture, non-hemiplegic side strength training, and structured home-based programs. Technology-assisted modalities included virtual reality balance training, robot-assisted gait training, and game-based balance training using the SBT-330 device. Cognitive–motor interventions included graded motor imagery, motor imagery therapy, action observation therapy, and Baduanjin exercise. Other modalities included horse-riding exercise, aquatic-based sequential preparatory training, and whole-body vibration combined with motor imagery.
- Duration and frequency: The intervention duration ranged from 3 to 4 weeks to 6 months. Session frequencies ranged from twice weekly to multiple daily sessions.
- Control conditions: Most studies (17/19) used a conventional rehabilitation control. Two studies used a self-before-after control design.
- Blinding: Blinding of participants and therapists was not feasible in any trial. Blinded outcome assessment was reported in 10 studies.

The detailed characteristics are presented in Table 3 Characteristics and details of the included studies. The mean age of the participants across the studies ranged from approximately 53 to 71 years, with males comprising 45% to 81% of the sample. Ischaemic stroke was more common than haemorrhagic stroke in most studies, accounting for 56% to 69% of cases reported. Among the studies that reported baseline MBI scores, the values ranged from approximately 35 to 88, the baseline BBS score ranged from approximately 11 to 42, and the baseline FMA-UE score ranged from approximately 15 to 32. The detailed baseline characteristics of each study are provided in Supplementary Table S3. The PEDro scores of the included studies ranged from 3 to 8 (median = 6). Six studies (32%) were classified as high quality (score ≥ 8), nine studies (47%) as moderate quality (score 6–7), and four studies (21%) as low quality (score ≤ 5). All the studies received a score of 0 on Items 5 and 6 because the blinding of participants and therapists is inherently infeasible in exercise intervention trials. Individual PEDro scores are provided in Supplementary Table S2.

**Table 3:** Characteristics and details of the included studies

Study	Sample (Exp/Con)	Outcomes	Duration and Frequency	Intervention	Core Intervention	Control	Control Details	Timing
1. Hongmei, 2022 [38]	80/80	FMA-UE, MBI, BBS	8 weeks, 15-minute separated finger induction training daily, once a day, 56 times total	Bilateral upper limb training	Bilateral upper limb synchronous training (passive joint movement, separated finger induction, bean bag throwing and catching, etc.)	Unilateral affected upper limb training	Only passive movement of the affected upper limb, muscle strength training and daily activity training	More than 1 month after stroke
2. Giulia Temperoni, 2020 [30]	18/15	MBI, TIS, BBS	4 weeks, 2 times/week, 45 min/session, 8 total sessions	Sequential water preparation training (SPA)	Sequential water training (kneeling posture → sitting posture → supine posture progression, combined with floating device gait preparation training)	Traditional water balance training	Traditional water training (warm-up, lower limb stretching, and walking practice)	More than 6 months after stroke
3. Hong-Guang Liu, 2024 [24]	50/50	FMA-UE, MBI	4 weeks, 5 times/week, 30 min/session, 20 total sessions	Acupuncture rehabilitation + upper limb task-oriented training	Acupuncture (scalp acupuncture + body acupuncture) + task-oriented training (touching targets, moving objects, and daily action simulation)	Scalp acupuncture + regular upper limb rehabilitation training	Scalp acupuncture + regular rehabilitation (Bobath technique, joint movement training, and functional electrical stimulation)	Acute stage of stroke (after the condition stabilized)
4. Ting He, 2025 [25]	18/18	FMA-UE, MBI	4 weeks, 5 times/week, 30 min/session, 20 total sessions	Virtual reality (VR) balance training	VR balance games (sunflower, space rescue, etc.), focusing on multidirection trunk control training	Traditional sitting balance training	Traditional balance training (push roller, stacking cups, plug-in board and other multidirection trunk activities)	Within 6 months after stroke (subacute stage)
5. Wenjun Jiang, 2021 [37]	19/20/18	FMA-UE, MBI, MSS, ARAT	4 weeks, once a day, 30 min/session, 5 days a week	Back group: healthy hand assists affected upper limb to place behind waist and straighten + regular rehabilitation + balance training. Shoulder elevation group: healthy hand assists the affected shoulder to elevate > 90° + regular rehabilitation + balance training	Control group: both arms naturally hang down on both sides of the trunk. Back group: the healthy hand assists the affected hand to place behind the waist, keeping the elbow joint straight. Shoulder elevation group: the healthy hand assists the affected shoulder to elevate > 90°, keeping the elbow joint straight	Control group: no upper limb intervention + regular rehabilitation + balance training	Fixed posture of the affected upper limb (without/behind–waist straight/shoulder elevation > 90° straight), and other variables are completely matched	Maintain the corresponding upper limb posture during daily balance training
6. Eun Kyu Ji, 2017 [34]	17/20	FMA-UE, MBI	8 weeks, 30 min/day (GMI) + regular rehabilitation	Regular rehabilitation + graded motor imagery (GMI) home training	GMI includes implicit motor imagery (left–right hand discrimination), explicit motor imagery (action simulation), mirror therapy	Regular rehabilitation training	Regular rehabilitation includes joint movement, muscle strength, fine motor training	Daily training at home
7. Pan Yingying, 2022 [27]	34/34	FMA-UE, MBI	6 months, daily (CIMT) + platform follow-up	Health platform continuous care + constraint-induced movement therapy (CIMT)	CIMT restricts the healthy upper limb (> 90% waking time), the affected limb undergoes stepping, balance and other training; the platform includes health education	Regular rehabilitation training	Regular rehabilitation includes standing, kneeling, and balance training (60 min/day)	Continuous intervention after discharge
8. Mingzhu Ye, 2022 [35]	24/24	FMA-UE, BBS	24 weeks, 40 min/session, 3 sessions/week (Baduanjin)	Health education + Baduanjin training	Follows the “Fitness Qigong Baduanjin Standard”, including 10 movements (preparation + 8 styles + closing)	Health education	Health education once a month (40 min/session)	Community centralized training
9. Jianming Fu, 2017 [28]	28/25	FMA-UE, MBI	8 weeks, 20 min/session, 6 sessions/week (action observation)	Regular rehabilitation + action observation therapy	Watch upper limb action videos (30 actions, divided into difficulty levels) + imitation training	Regular rehabilitation treatment	Regular rehabilitation includes Bobath and Brunnstrom techniques and daily activity training	Daily intervention
10. Wan Liu, 2024 [22]	8/12	FMA-UE, MBI	4 weeks, 2 h/day, 5 days/week (CRT) + 30 min/session (MIT)	Regular rehabilitation treatment (CRT) + motor imagery therapy (MIT)	MIT includes motor schema, perception integration, body intention training, focusing on mirror imitation	Regular rehabilitation treatment (CRT)	CRT includes physical therapy, occupational therapy, electrical stimulation, and acupuncture	Daily intervention
11. Ryan E. Ross, 2023 [36]	10/10	FMA-UE, MoCA	18 sessions, 15 min/session (AEx) + 20 min/session (VR - UE), 2–3 times/week	Lower limb aerobic training (AEx) + virtual reality upper limb rehabilitation (VR-UE)	AEx is a recumbent bicycle (70% heart rate reserve); VR-UE is a duck punch game (forwards extension action training)	No separate control group (single-group feasibility test)	Before-and-after baseline self-control	Each intervention starts with AEx followed by VR
12. Can Ao, 2025 [29]	72/67	FMA-UE, MBI, BBS, ARAT	3 months, MI: 5 days/week; WBV: 6 days/week	MI + WBV therapy	MI: 120 cm × 60 cm mirror, 10 daily action videos (played 3 times); WBV: vibration	Traditional rehabilitation training	Traditional rehabilitation training: exercise therapy + occupational therapy + standing training + deep squat exercise (45 min/session, 3 sessions/day)	MI: continuous throughout; WBV: continuous throughout MI: continuous throughout; WBV: continuous throughout
13. Xiao-Jun Yin, 2021 [33]	16/16	FMA-UE, BBS, HAMD	6 weeks, 20 min/session, 1 session/day	MIT: audio-guided lower limb action imagination	MIT: 3 min of relaxation + 15 min of lower limb action imagination (each action repeated 3 times), audio-guided	Regular neurological rehabilitation	Regular rehabilitation: 3 h/session, 5 sessions/week, the intensity was adjusted according to the functional status	Fixed time period in the afternoon
14. Yen-Nung Lin, 2022 [31]	20/20	FMA-UE, BBS	3–4 weeks, 30 min/session, 5 sessions/week (15 total sessions)	Regular rehabilitation + RAGT: hybrid robot gait training	RAGT: elliptical trajectory gait training, step length/speed gradually increase (maximum of 100% step length, speed of 0.917 km/h)	Regular rehabilitation: transfer training + balance training + walking training	Regular rehabilitation: 130 min/session, including personalized balance and walking training	After regular rehabilitation during hospitalization
15. Jung-Ho Lee, 2023 [32]	14/14	MBI, TIS, BBS	6 weeks, 30 min/session, 5 sessions/week (30 total sessions)	Regular rehabilitation + neurological rehabilitation diagonal training using the PNF technique	Diagonal training: PNF D1/D2 mode, upper and lower limb diagonal actions (10 times/group × 3 groups, 1-minute rest between groups)	Regular rehabilitation + sagittal plane movement training	Sagittal plane training: dynamometer training, initial intensity at 20% of the heart rate reserve, 5-minute warm-up/recovery	After regular rehabilitation
16. Yong-Nam Kim, 2015 [23]	10/10	MBI, BBS	6 weeks, 30 min/session, 5 days/week	Horse-riding movement	Horse-riding equipment: three-dimensional actions (twisting, sliding, and rolling), full-course/local courses alternate, progression through difficulty levels 1–4	Neurodevelopmental therapy (NDT)	NDT method: 30 min/session, the intensity is adjusted according to the patient’s tolerance	Course of disease ≥ 6 months, after the condition stabilizes
17. Jung-Lim Lee, 2024 [39]	19/14	FMA-UE, MBI, TIS, BBS	4 weeks, 30 min/session, 5 sessions/week	Regular physical therapy + SBT-330 balance training	SBT-330: standing posture weight sensor feedback, fruit catching + archery games (15 min each), centre-of-gravity transfer control cursor/aim	Regular physical therapy	Regular physical therapy: 30 min/session, alternating lower limb strengthening, function training, and gait training	Course of disease ≤ 24 months, BBS score of 21–40 points
18. Chenlan Shao, 2022 [21]	63/64	MBI, BBS	6 weeks, 45 min/session, 5 days/week	Regular rehabilitation + non-hemiplegic side (NHS) strength training	Nonhemiplegic side strength training: resistance band stepping (10–15 times/group × 3 groups) + pulling training (10–15 times/group × 3 groups)	Regular rehabilitation training	Regular rehabilitation: hemiplegic side stepping + obstacle crossing (10–15 times/group × 3 groups each) + standing training	Course of disease ≤ 6 weeks, after the first stroke
19. Yang-Chool Lee, 2015 [26]	12/0	MBI, BBS	8 weeks, ≥ 5 times a day, approximately 10 min per session, ≥ 5 days a week	Self-bedside exercise	Whole-body movement: shoulder stretching, waist relaxation, etc.; fine movement: knocking, grasping, etc.; movement coordination such as standing, hip training, etc.	No separate control group (before-and-after self-control)	Before-and-after baseline self-control	During hospitalization, self-performed daily

*Abbreviations*: *FMA-UE*, Fugl–Meyer Assessment of Upper Extremity motor function; *MBI*, modified Barthel index; BBS, Berg Balance Scale; *TIS*, Trunk Impairment Scale; *MSS*, Motor Status Scale;* ARAT*, Action Research Arm Test; *MoCA*, Montreal Cognitive Assessment; *HAMD*, Hamilton Depression Rating Scale; *GMI*, graded motor imagery; *CIMT*, constraint-induced movement therapy; *CRT*, conventional rehabilitation training; *AEx*, aerobic exercise; *VR*, virtual reality; *WBV*, whole-body vibration; *PNF*, proprioceptive neuromuscular facilitation; *RAGT*, robot-assisted gait training; *NDT*, neurodevelopmental therapy; *NHS*, nonhemiplegic side

### Results of the meta-analysis

The overall MBI, BBS and FMA-UE scores from 19 studies were summarized, and a meta-analysis was conducted using a random effects model. The results showed that exercise interventions improved the MBI (SMD = 0.95, 95% CI: 0.42–1.49, *p* = 0.0004), BBS (SMD = 0.73, 95% CI: 0.24–1.22, *p* = 0.003) and FMA-UE (SMD = 0.62, 95% CI: 0.16–1.07, *p* = 0.008) scores. However, moderate to high heterogeneity was observed for each outcome measure (for details, see the specific analysis of each outcome), and the assessment of publication bias is shown in the funnel plots corresponding to each outcome.

### MBI scores

**Fig. 5:**
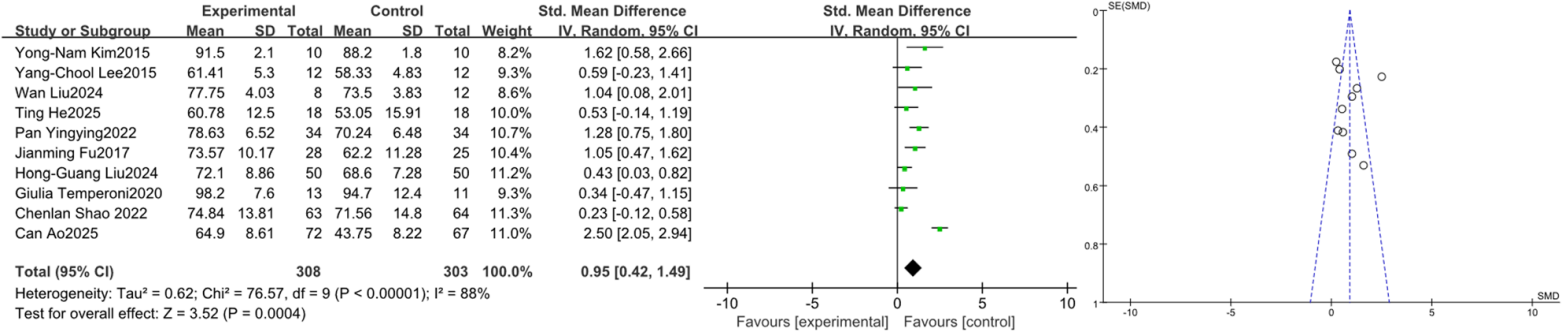
Main analysis of the MBI score

A total of 10 studies (sample size *n* = 611, 308 patients in the experimental group and 303 patients in the control group) reported modified Barthel index scores. The meta-analysis revealed that exercise interventions can improve the MBI score of stroke patients and has clinical reference value. The combined standardized mean difference was 0.95 (95% CI: 0.42–1.49, *P* = 0.0004), and extremely high heterogeneity was observed among the studies (I²=88%, *P* < 0.00001). The funnel plot shows a low risk of publication bias. Existing studies have relatively comprehensive collections, but due to the extremely high heterogeneity (I²=88%), the conclusions need to be interpreted cautiously (Fig. 5).

### Main analysis of the MBI scores

**Fig. 6:**
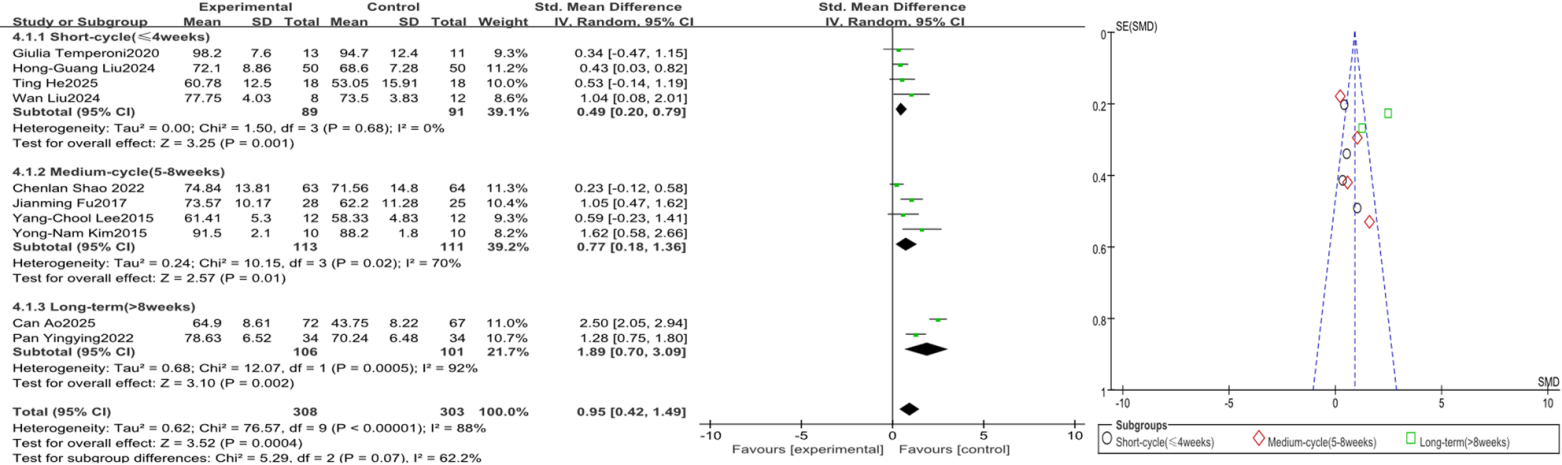
Subgroup analysis of the MBI scores of patients stratified by the intervention cycle

The subgroup analysis based on the intervention periods revealed that all the exercise interventions in each period improved the MBI score, and the effect size tended to increase as the duration of the intervention increased (≤ 4 weeks: SMD = 0.49, 95% CI: 0.20–0.79, P = 0.001; 5–8 weeks: SMD = 0.77, 95% CI: 0.18–1.36, P = 0.01; > 8 weeks: SMD = 1.89, 95% CI: 0.70–3.09, P = 0.002). No statistically significant difference was observed among the subgroups (P = 0.07). The funnel plot shows that the distribution of scattered points in each subgroup is essentially symmetrical, indicating a low risk of publication bias Fig. 6.

BBS Scores

### BBS scores

**Fig. 7:**
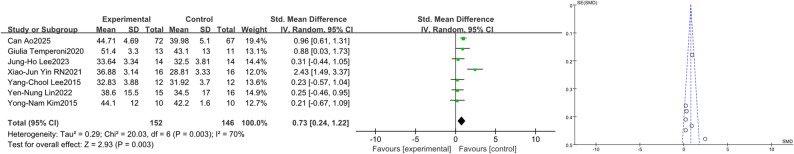
Main analysis of the BBS score

A total of 7 studies (sample size n = 298) reported BBS scores. The meta-analysis revealed that exercise interventions improved the BBS score of stroke patients, with a combined standardized mean difference of 0.73 (95% CI: 0.24–1.22, P = 0.003). Moderate to high heterogeneity was observed among the studies (I² = 70%, P = 0.003). The funnel plot shows a relatively dispersed distribution of the BBS score. Due to the medium to high heterogeneity (I²=70%), caution should be exercised when interpreting the results, and a clear determination of the risk of publication bias is currently impossible (Fig. 7).

**Fig. 8:**
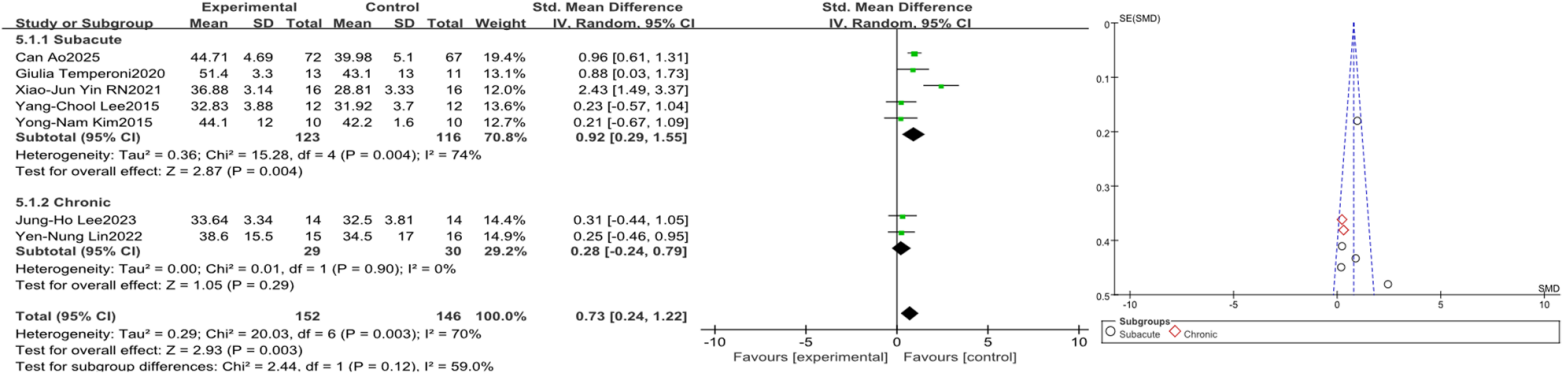
Subgroup analysis of the BBS scores of patients stratified by disease stage

The subgroup analysis based on the disease stage revealed that the effect was significant in the subacute stage (SMD = 0.92, 95% CI: 0.29–1.55, P = 0.004), whereas the effect was weaker in the chronic stage and did not reach statistical significance (SMD = 0.31, 95% CI: -0.27–0.89, P = 0.29), and the differences between the subgroups were not statistically significant (P = 0.12). Only 2 studies included patients in the chronic stage, and these results should be interpreted with caution. A scatter plot of the subacute group from the funnel plot can be constructed, but assessing the symmetry of the chronic group is difficult because of the small number of studies, and the risk of publication bias is still unclear (Fig. 8).

### Overall FMA-UE scores

**Fig. 9:**
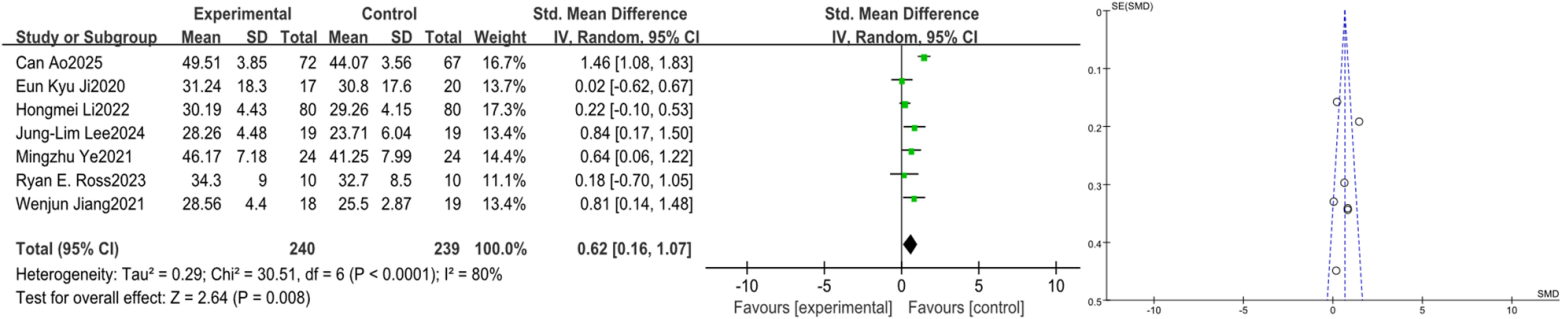
Main analysis of the FMA-UE score

A total of 7 studies (sample size n = 479) reported FMA-UE scores. The meta-analysis revealed that exercise interventions improved the FMA-UE scores of stroke patients, and the combined standardized mean difference was 0.62 (95% CI: 0.16–1.07, P = 0.008). High heterogeneity was detected among the studies (I² = 80%, P < 0.0001). The funnel plot shows a discrete distribution. Due to high heterogeneity (I²=80%), caution should be exercised in the interpretation of the results, and the risk of publication bias cannot be clearly determined for the time being (Fig. 9).

**Fig. 10:**
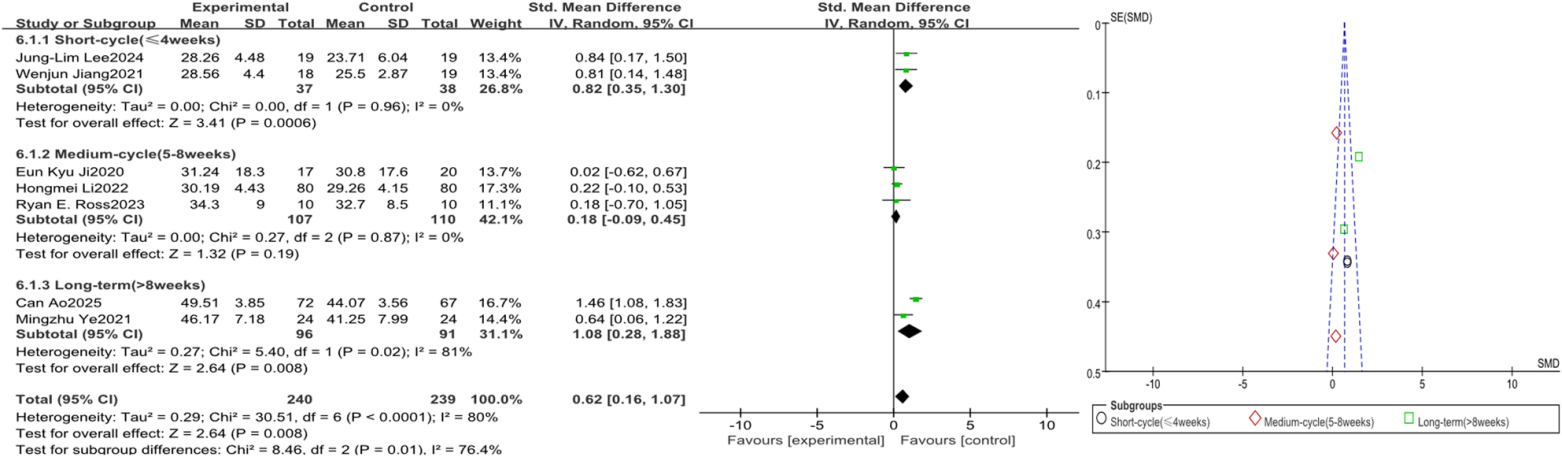
Subgroup analysis of the FMA-UE scores of patients stratified by the intervention cycle

The subgroup analysis by intervention period revealed that the duration of intervention had a significant regulatory effect on the recovery of upper limb function. The short-term group (≤ 4 weeks, 2 items) showed a significant effect (SMD = 0.82, 95% CI: 0.35–1.30, P = 0.0006), the medium-term group (5–8 weeks, 3 items) showed a smaller and nonsignificant effect (SMD = 0.18, 95% CI: -0.09–0.45, P = 0.19), and the long-term group (> 8 weeks, 2 items) exhibited the greatest effect (SMD = 1.08, 95% CI: 0.28–1.88, P = 0.008). A significant difference was observed among the subgroups (P = 0.01), indicating that the intervention period was a significant factor regulating the recovery of upper limb function, which was consistent with the dose–effect relationship revealed by the subsequent meta-regression analysis. The funnel plot showed that the number of studies in each subgroup was limited (2–3 items), the scatter distribution did not show obvious asymmetry, but the test power was insufficient, and the risk of publication bias was difficult to clearly determine (Fig. 10).

### Sensitivity analysis and extended subgroup analyses

Sensitivity analyses: The leave-one-out analysis supported the stability of the primary results. For the MBI score, the pooled SMD after removing any single study ranged from 0.72 to 1.05, with all P values remaining less than 0.01. For the BBS score, the range was 0.56 to 0.82 (*P* < 0.05 throughout), and for the FMA-UE score, the range was 0.41 to 0.71 (*P* < 0.05 throughout). No single study altered the direction or significance of the pooled estimates. After excluding studies with a PEDro score less than 6, the pooled SMD changed from 0.95 to 0.58 for the MBI score, from 0.73 to 0.79 for the BBS score, and from 0.62 to 0.45 for the FMA-UE score. All three outcomes remained statistically significant (MBI score, *P* = 0.0007; BBS score, *P* = 0.04; FMA-UE score, *P* = 0.004). Detailed results of the leave-one-out and quality-based sensitivity analyses are provided in Supplementary Tables S1 and S2.

Extended subgroup analyses: When the patients were stratified by stroke stage, all the subgroups experienced favourable effects of exercise. A pooled SMD of 0.71 was obtained for the MBI score of the subacute group, and the SMD was 1.28 for the chronic group, with no significant between-group difference (*P* = 0.36). With respect to the FMA-UE score, the SMDs of the subacute and chronic groups were 0.50 and 0.72, respectively, again without a significant difference across the subgroups (*P* = 0.67). Comparable findings for the BBS score were reported in the main subgroup analyses.

When the data were stratified by exercise type, the pooled SMDs were consistently positive across categories. For the MBI score, the SMDs of the conventional, technology-assisted, and mind-body groups were 0.73, 1.38, and 0.64, respectively, with no significant subgroup effect (*P* = 0.37). For the BBS score, the corresponding SMDs were 0.23, 0.93, and 1.30 (*P* = 0.18 for the difference between subgroups). For the FMA-UE score, the conventional group contained only one study and was not analysed as a separate subgroup; the technology-assisted and mind-body groups had SMDs of 0.91 and 0.49, respectively, with no significant difference between subgroups (*P* = 0.19). Given that some of these subgroups included very few studies, the results should be interpreted with caution. The corresponding plots are shown in Supplementary Figures S2 through S6. The GRADE assessment rated the certainty of evidence as very low for all three primary outcomes, primarily because of high heterogeneity, a risk of bias in the included trials, and limited numbers of studies.

### Risk of bias and publication bias assessments

Most studies showed a low risk of bias in the randomization process, but due to the particularity of exercise interventions, participant and intervener blinding is difficult to implement, and a certain risk exists in the field of “outcome measurement”. Extremely high heterogeneity was observed for the MBI and FMA-UE scores, which may mask potential bias. The interpretation of these conclusions requires caution.

3.6 Statistics for the Meta-Regression Model.

The meta-regression model statistics are presented in Table 4 Statistics. The R² for the model was 0.762, indicating that the total dose explained approximately 76% of the variance in FMA-UE effect sizes. The dose coefficient was statistically significant (β₁ = 0.0003, SE = 0.000081, t = 3.71, df = 5, *P* = 0.014), with a 95% CI of (0.00009, 0.00051). The quadratic term was not significant, supporting the linear model for the current data. All Cook’s D values were less than 1, suggesting the absence of influential outliers and acceptable stability of the model.

**Table 4:** Statistics for the meta-regression model

Statistic	Value
R²	0.762
β_1_	0.0003
SE(β_1_)	0.000081
t (df = 5)	3.71
P value	0.014
95% CI	(0.00009, 0.00051)

## Discussion

### Efficacy of exercise interventions on core poststroke functions

This systematic review and meta-analysis synthesized evidence from 19 randomized controlled trials. Our findings indicate that exercise interventions consistently and significantly improve balance (BBS), activities of daily living (MBI), and upper extremity motor function (FMA-UE) among stroke survivors. The pooled effect sizes for the MBI (SMD = 0.95, 95% CI: 0.42–1.49, *p* = 0.0004), BBS (SMD = 0.73, 95% CI: 0.24–1.22, *p* = 0.003), and FMA-UE (SMD = 0.62, 95% CI: 0.16–1.07, *p* = 0.008) scores were all statistically significant and exhibited robust consistency. These results provide updated, evidence-based support reaffirming that exercise is a cornerstone of stroke rehabilitation [40].

### The “193-hour concept”: an exploratory dose–response analysis

The main analysis confirmed a positive effect of exercise on the FMA-UE score, but the high heterogeneity across studies suggested that the benefit might depend on certain factors [41]. We examined the relationship between the cumulative exercise dose and improvement in the FMA-UE score by performing a meta-regression analysis to answer the practical question of how much exercise is needed to produce a clinically meaningful change. A linear relationship was observed, and the dose corresponding to an MCID of 5 points was approximately 193 h, which we term the “193-hour theory [42].” 

Based on the available data, this threshold corresponds approximately to the exercise dose at which the mean improvement in the FMA-UE score reaches the MCID of 5 points. Below this level, the observed gains may not reach clinical significance. Above this level, further improvement is possible but appears to diminish, and excessive dosing may increase the risk of fatigue or joint strain. Given the small number of studies available for this analysis (*n* = 7), this 193-hour estimate should be regarded as a preliminary observation. Whether it represents a true minimum effective dose requires testing in prospective studies.

In practice, this threshold has three useful features. If confirmed, it would provide a specific, cumulative time target rather than relying on vague advice about adequate exercise. Second, it is reported in hours and minutes and can be discussed and tested in clinical settings, although it is not yet a validated prescription tool. Third, as an approximate minimum rather than a rigid prescription, it can be adjusted for the characteristics of individual patients, although the appropriate range of adjustment remains to be determined [43].

### The mechanism by which exercise interventions improve the function of stroke patients

The effects of exercise on improving the MBI, BBS and FMA-UE scores involve multiple aspects, such as behavioural adaptation and neurophysiological changes. The improvement in the MBI score directly reflects the recovery of activities of daily living (such as eating and dressing) because exercise training can effectively increase the core muscle strength, overall endurance and limb coordination of patients [44]. The improvement in the BBS score is more specifically related to the optimization of the posture control system by movement, including increasing trunk stability; improving the efficiency of centre of gravity transfer; and integrating visual, vestibular and proprioceptive inputs, thereby reducing the risk of falls [45]. The improvement in the fine motor function of the upper limbs evaluated by the FMA-UE most profoundly reflects motor-induced neural plasticity [46]. Repetitive and task-specific upper limb training (whether active movement, mirror therapy or robot-assisted training) can promote the reorganization of the sensorimotor cortex of the brain; strengthen the synaptic connections of damaged pathways; and may improve muscle activation timing, joint separation movement and grasp coordination through mechanisms such as the activation of the mirror neuron system and compensation by the contralateral hemisphere. Therefore, exercise interventions not only affect the peripheral musculoskeletal system but also provide a fundamental impetus for functional recovery through adaptive changes in the central nervous system [47].

### Clinical and policy implications of exercise interventions in stroke rehabilitation

The results of this study provide evidence for the systematic integration of structured exercise interventions into the clinical pathway of stroke rehabilitation. Clinical workers should prioritize the selection of exercise programs supported by targeted evidence based on the patient’s main functional impairment (balance, daily activities or upper limb function) and fully consider the stage of stroke: in the acute/subacute stage, task-oriented training can be emphasized to quickly establish a basic exercise pattern [48]; during the chronic phase, more challenging training that integrates life scenarios should be designed to break through the functional platform [49]. At the level of health policy and rehabilitation management, efforts should focus on (1) formulating and promoting evidence-based operation guidelines for exercise rehabilitation; (2) incorporating low-cost and easily scalable exercise intervention programs into the community rehabilitation service system and long-term care plans to increase the accessibility and continuity of rehabilitation services [50]; and (3) strengthening the training of the rehabilitation team to ensure the scientific implementation and monitoring of the safety of exercise prescriptions.

### Advantages and limitations of this study

This study was strengthened by adherence to the PRISMA guidelines, a comprehensive search strategy, independent screening and data extraction, and dual-quality assessment using RoB2 and PEDro. However, several limitations should be noted.

- The number of included RCTs was limited, and a large proportion of full-text articles could not be retrieved, which may have introduced selection bias
- Considerable clinical and methodological heterogeneity existed across exercise modalities, stroke stages, and assessment timing
- Subgroup and meta-regression analyses were based on small numbers of studies, and the dose–response estimate should therefore be considered exploratory
- Publication bias could not be fully excluded, and the GRADE assessment indicated very low certainty of evidence for all outcomes

Therefore, the findings should be interpreted with caution.

### Future research directions and prospects

Future research should address the following directions:

- Large-scale RCTs with blinded outcome assessments should be conducted to compare different exercise modalities and intensities
- The exercise intensity, frequency, duration, and total dose should be reported in detail to enable more precise dose‒response analyses [51]
- These studies should include chronic-phase patients and those with severe impairments, who are underrepresented in current trials
- The effectiveness and cost-effectiveness of technology-assisted interventions such as VR and robot-assisted training should be evaluated in community and home-based settings [52]
- The barriers to and facilitators of the implementation of structured exercise programs in diverse clinical and community settings should be investigated [53]

## Conclusions

This systematic review and meta-analysis of 19 RCTs involving 1,041 stroke patients confirmed that structured exercise significantly improves balance, activities of daily living, and upper limb motor function. The meta-regression analysis revealed a linear dose‒response relationship between the cumulative exercise dose and the improvement in the FMA-UE score, from which the “193-hour theory” is proposed: approximately 193 h of structured exercise is needed to achieve clinically meaningful upper limb recovery, as defined by the minimal clinically important difference of 5 points in the FMA-UE score. These exploratory findings suggest an approximate dose target of 193 h, which may serve as a preliminary reference for an exercise prescription, pending prospective validation.

## Supplementary Information

Supplementary Material 1.

Supplementary Material 2.

## Data Availability

All data generated or analyzed during this study are included in this published article and its supplementary information files. The datasets used and/or analyzed during the current study are available from the corresponding author on reasonable request.

## Abbreviations

MBI: modified Barthel index
BBS: Berg Balance Scale
FMA-UE: Fugl–Meyer Assessment of Upper Extremity motor function
RCT: randomized controlled trial
PRISMA: Preferred Reporting Items for Systematic Reviews and Meta-Analyses
MCID: minimal clinically important difference
