# Supplementary material for: Exercise intervention improves the modified Barthel index, Berg balance scale, and Fugl–Meyer assessment of upper limb motor function in stroke patients: a systematic review and meta-analysis
Source: BMC Sports Sci Med Rehabil. 2026 May 22;18:329. doi: 10.1186/s13102-026-01764-z (PMC13374158; doi:10.1186/s13102-026-01764-z)
Supplement: Supplementary file 2 — Supplementary Material 2. [file 13102_2026_1764_MOESM2_ESM.docx]

# Supplementary materials

## Non-standard abbreviations and acronyms

MBI Modified Barthel Index

BBS Berg Balance Scale

FMA-UE Fugl-Meyer Upper Limb Motor Function Assessment Scale

## MBI Data Sheet


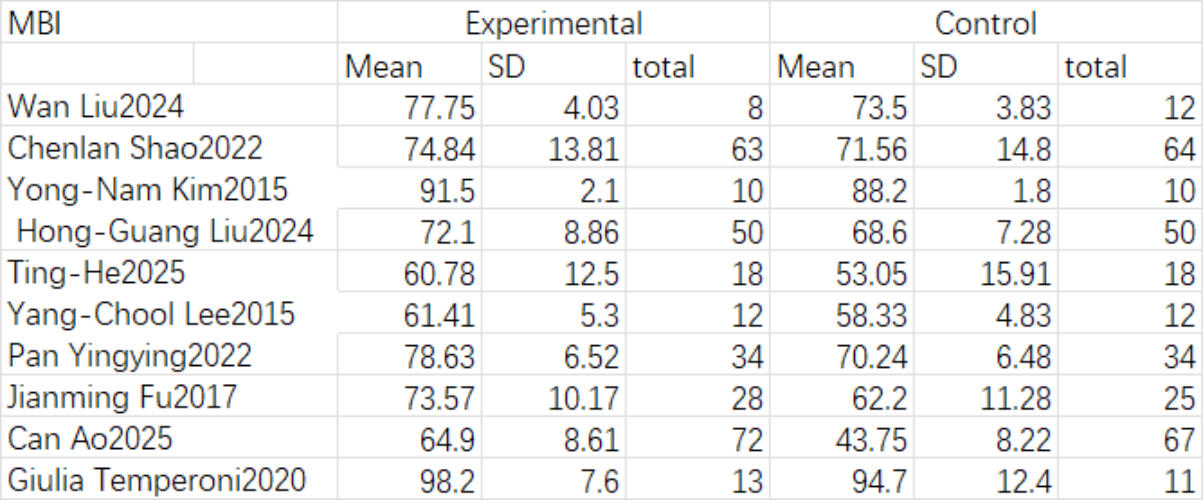


## BBS Data Sheet


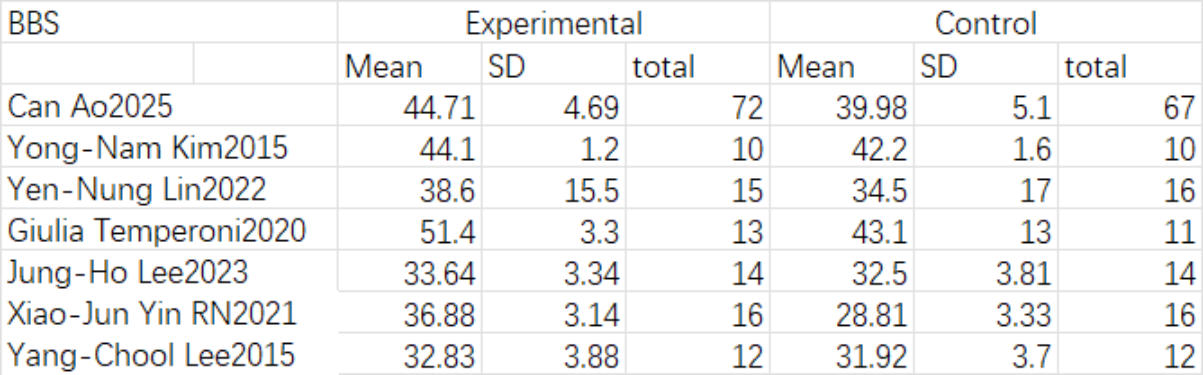


## FMA-UE Data Sheet


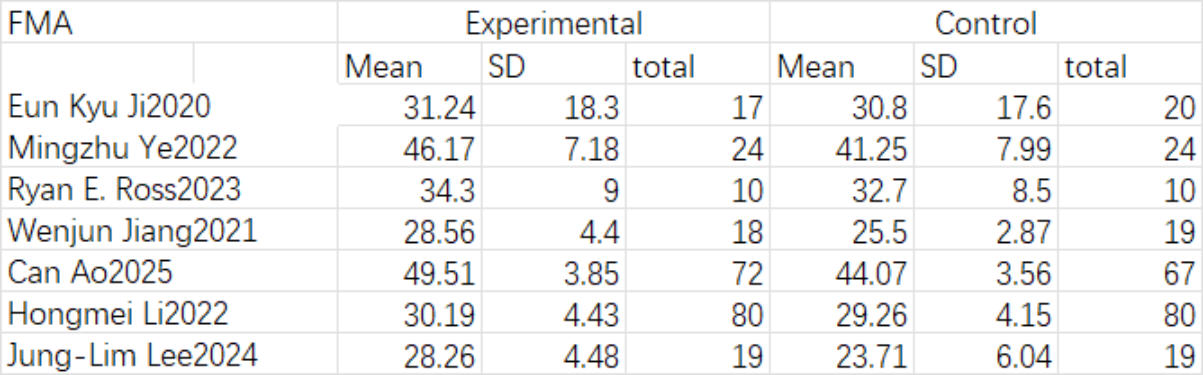


## MBI Subgroup Analysis Data Sheet


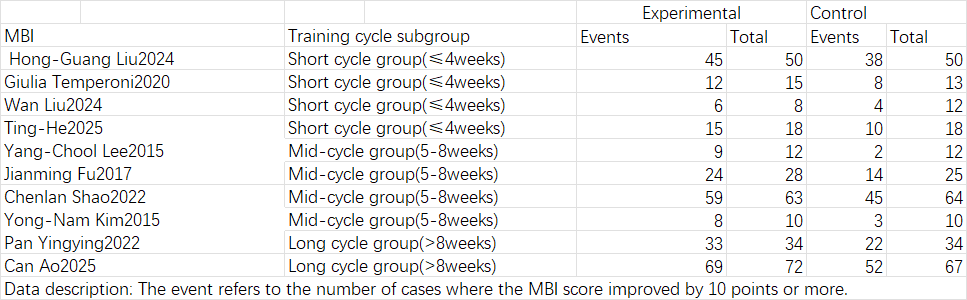


## BBS Subgroup Analysis Data Sheet


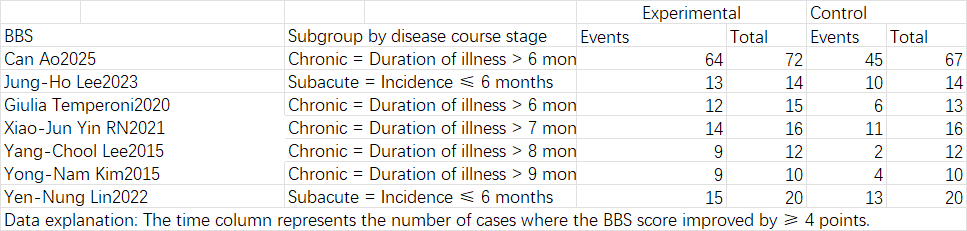


## FMA-UE Subgroup Analysis Data Sheet


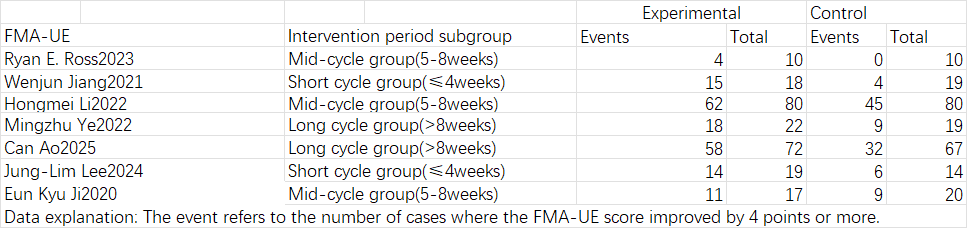


Fig S1 Abstract-level screening of unobtainable full texts


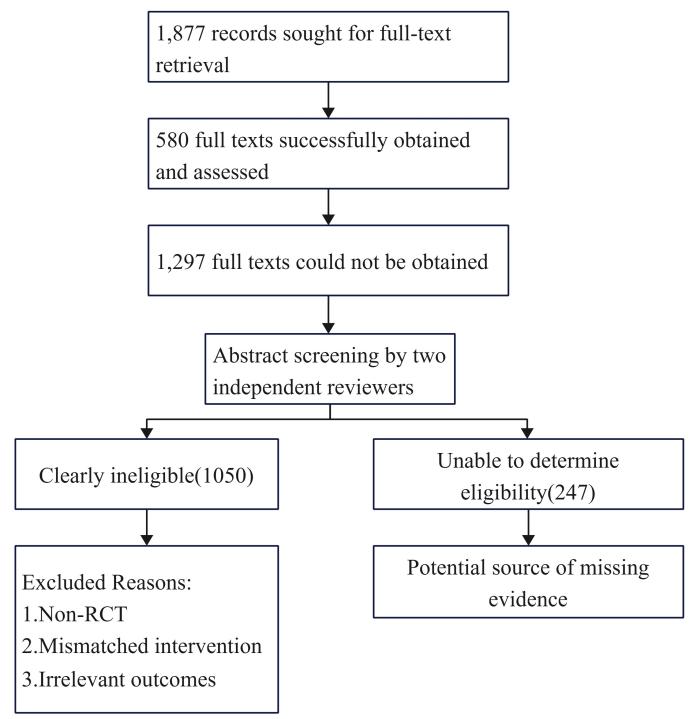


Fig. S1. Abstract-level screening of unobtainable full texts. Of 1,877 records sought for full-text retrieval, 580 were successfully obtained and assessed, while 1,297 could not be obtained. Two independent reviewers screened the abstracts of all 1,297 records. Approximately 1,050 (81%) were clearly ineligible (non-RCT, mismatched intervention, or irrelevant outcomes). The remaining approximately 247 (19%) had insufficient abstract information to determine eligibility and represent a potential source of missing evidence.

Supplementary Table S1 PEDro scores of the included studies.

| Study | Item 2 | Item 3 | Item 4 | Item 5 | Item 6 | Item 7 | Item 8 | Item 9 | Item 10 | Item 11 | Total (0–10) |
| --- | --- | --- | --- | --- | --- | --- | --- | --- | --- | --- | --- |
| Hongmei Li 2022 | 1 | 0 | 1 | 0 | 0 | 0 | 1 | 1 | 1 | 1 | 6 |
| Giulia Temperoni 2020 | 1 | 1 | 1 | 0 | 0 | 1 | 1 | 0 | 1 | 1 | 7 |
| Hong-Guang Liu 2024 | 1 | 0 | 1 | 0 | 0 | 0 | 1 | 1 | 1 | 1 | 6 |
| Ting He 2025 | 1 | 1 | 1 | 0 | 0 | 1 | 1 | 1 | 1 | 1 | 8 |
| Wenjun Jiang 2021 | 1 | 0 | 1 | 0 | 0 | 1 | 1 | 1 | 1 | 1 | 7 |
| Eun Kyu Ji 2020 | 1 | 0 | 1 | 0 | 0 | 0 | 1 | 1 | 1 | 1 | 6 |
| Pan Yingying 2022 | 0 | 0 | 1 | 0 | 0 | 0 | 1 | 1 | 1 | 1 | 5 |
| Mingzhu Ye 2022 | 1 | 1 | 1 | 0 | 0 | 1 | 1 | 1 | 1 | 1 | 8 |
| Jianming Fu 2017 | 1 | 0 | 1 | 0 | 0 | 0 | 1 | 1 | 1 | 1 | 6 |
| Wan Liu 2024 | 1 | 0 | 1 | 0 | 0 | 1 | 0 | 0 | 1 | 1 | 5 |
| Ryan E. Ross 2023 | 0 | 0 | 1 | 0 | 0 | 0 | 1 | 1 | 0 | 1 | 4 |
| Can Ao 2025 | 0 | 0 | 1 | 0 | 0 | 0 | 1 | 1 | 1 | 1 | 5 |
| Xiao-Jun Yin 2021 | 1 | 1 | 1 | 0 | 0 | 1 | 1 | 1 | 1 | 1 | 8 |
| Yen-Nung Lin 2022 | 1 | 1 | 1 | 0 | 0 | 1 | 1 | 1 | 1 | 1 | 8 |
| Jung-Ho Lee 2023 | 0 | 0 | 1 | 0 | 0 | 1 | 1 | 1 | 1 | 1 | 6 |
| Yong-Nam Kim 2015 | 1 | 0 | 1 | 0 | 0 | 0 | 1 | 1 | 1 | 1 | 6 |
| Jung-Lim Lee 2024 | 1 | 0 | 1 | 0 | 0 | 1 | 1 | 1 | 1 | 1 | 7 |
| Chenlan Shao 2022 | 1 | 1 | 1 | 0 | 0 | 1 | 1 | 1 | 1 | 1 | 8 |
| Yang-Chool Lee 2015 | 0 | 0 | 1 | 0 | 0 | 0 | 1 | 0 | 0 | 1 | 3 |
| Count (%) | 15 (79%) | 6 (32%) | 19 (100%) | 0 (0%) | 0 (0%) | 10 (53%) | 18 (95%) | 16 (84%) | 18 (95%) | 19 (100%) |  |

Supplementary Table S2 Baseline characteristics of the included studies

| Study | Mean Age (yr) | Sex (M/F) | Stroke Type (I/H) | Baseline MBI | Baseline BBS | Baseline FMA-UE |
| --- | --- | --- | --- | --- | --- | --- |
| Hongmei Li 2022 | 59.0 | 84/76 | — | 38.1 ± 6.2 | — | 15.2 ± 3.3 |
| Giulia Temperoni 2020 | 52.2 | 21/12 | 13/7 | 85.0 ± 10.9 | 38.8 ± 9.1 | — |
| Hong-Guang Liu 2024 | 61.2 | 57/43 | 32/18 | 40.7 ± 6.5 | — | 28.9 ± 3.1 |
| Ting He 2025 | 61.6 | 27/9 | 13/10 | 56.9 ± 14.5 | — | — |
| Wenjun Jiang 2021 | 56.5 | 30/27 | 33/24 | 80.8 ± 8.3 | — | 24.1 ± 3.7 |
| Eun Kyu Ji 2020 | 57.5 | 22/15 | 16/21 | 69.2 ± 22.2 | — | 27.7 ± 17.4 |
| Pan Yingying 2022 | 53.5 | 37/31 | 31/37 | 62.2 ± 8.7 | — | 23.5 ± 8.6 |
| Mingzhu Ye 2022 | 62.2 | 41/7 | 25/23 | — | 40.0 ± 3.8 | 56.0 ± 10.5 |
| Jianming Fu 2017 | 61.0 | 22/31 | I only | 55.9 ± 11.1 | — | 30.5 ± 11.5 |
| Wan Liu 2024 | 59.4 | 11/9 | 12/8 | 60.0 ± 4.1 | — | 17.3 ± 2.3 |
| Ryan E. Ross 2023 | 53.6 | 6/4 | 8/2 | — | — | 32.7 ± 8.5 |
| Can Ao 2025 | 70.9 | 81/58 | 83/56 | 46.2 ± 6.1 | 20.2 ± 5.7 | 34.1 ± 4.3 |
| Xiao-Jun Yin 2021 | 57.0 | 25/7 | 21/11 | — | 11.4 ± 2.8 | 14.6 ± 2.3 |
| Yen-Nung Lin 2022 | 55.3 | 11/29 | 28/12 | 31.2 ± 16.3 | — | 7.6 ± 4.8 |
| Jung-Ho Lee 2023 | 67.6 | — | I only | 34.7 ± 5.7 | 28.6 ± 3.9 | — |
| Yong-Nam Kim 2015 | 70.2 | 10/10 | — | 87.7 ± 1.7 | 42.0 ± 1.3 | — |
| Jung-Lim Lee 2024 | 64.5 | 21/12 | — | 58.3 ± 13.5 | 34.1 ± 5.7 | 20.4 ± 6.1 |
| Chenlan Shao 2022 | 65.1 | 84/43 | 79/48 | 35.6 ± 5.8 | 24.2 ± 5.3 | — |
| Yang-Chool Lee 2015 | — | — | — | 58.3 ± 4.8 | 31.9 ± 3.7 | — |

Table S2:The missing values in the baseline characteristics table reflect the limitations of the original literature reports. The items marked with “—” confirm that they were not reported in the original text. Each study only measured the baseline values of its pre-specified outcome indicators, so not all studies reported MBI, BBS, and FMA-UE. Some studies did not stratify patient characteristics by gender or stroke subtype. These missing values have been truthfully reflected in the table and do not require additional processing.

Table S3 Leave-one-out sensitivity analysis for MBI

| The removed study | Remaining merged SMD | 95%Cl | P |
| --- | --- | --- | --- |
| MBI | 0.95 | 0.42-1.49 | 0.0004 |
| Can Ao 2025 | 0.72 | 0.41-1.03 | <0.00001 |
| Chenlan Shao 2022 | 1.05 | 0.49-1.61 | 0.0003 |
| Giulia Temperoni 2020 | 1.02 | 0.45-1.59 | 0.0005 |
| Hong-Guang Liu 2024 | 1.02 | 0.42-1.62 | 0.0009 |
| Jianming Fu 2017 | 0.94 | 0.35-1.54 | 0.002 |
| Pan Yingying2022 | 0.92 | 0.32-1.51 | 0.002 |
| Ting He 2025 | 1.00 | 0.42-1.58 | 0.0007 |
| Wan Liu 2024 | 0.95 | 0.38-1.52 | 0.001 |
| Yang-Chool Lee 2015 | 0.99 | 0.42-1.57 | 0.0007 |
| Yong-Nam Kim 2015 | 0.89 | 0.33-1.45 | 0.002 |

Table S4 Leave-one-out sensitivity analysis for BBS

| The removed study | Remaining merged SMD | 95%Cl | P |
| --- | --- | --- | --- |
| BBS | 0.73 | 0.24-1.22 | 0.003 |
| Can Ao 2025 | 0.69 | 0.06-1.32 | 0.03 |
| Giulia Temperoni 2020 | 0.71 | 0.15-1.28 | 0.01 |
| Jung-Ho Lee 2023 | 0.80 | 0.25-1.36 | 0.004 |
| Xiao-Jun Yin RN 2021 | 0.56 | 0.23-0.90 | 0.0009 |
| Yang-Chool Lee 2015 | 0.81 | 0.27-1.36 | 0.003 |
| Yen-Nung Lin 2022 | 0.82 | 0.27-1.37 | 0.003 |
| Yong-Nam Kim 2015 | 0.81 | 0.27-1.35 | 0.003 |

Table S5 Leave-one-out sensitivity analysis for FMA-UE

| The removed study | Remaining merged SMD | 95%Cl | P |
| --- | --- | --- | --- |
| FMA-UE | 0.62 | 0.16-1.07 | 0.008 |
| Can Ao 2025 | 0.41 | 0.14-0.68 | 0.003 |
| Eun Kyu Ji 2020 | 0.71 | 0.22-1.20 | 0.005 |
| Hongmei Li 2022 | 0.70 | 0.22-1.19 | 0.004 |
| Jung-Lim Lee 2024 | 0.58 | 0.06-1.10 | 0.03 |
| Mingzhu Ye 2022 | 0.61 | 0.07-1.14 | 0.03 |
| Ryan E. Ross 2023 | 0.67 | 0.17-1.17 | 0.008 |
| Wenjun Jiang 2021 | 0.58 | 0.06-1.10 | 0.03 |

Table S6 Quality-based sensitivity analysis: pooled SMD before and after excluding studies with PEDro < 6

|  | All 10 studies | Excluding PEDro < 6 |
| --- | --- | --- |
| MBI |  |  |
| Remaining merged SMD | 0.95 | 0.58 |
| P | 0.0004 | 0.0007 |
| 95%Cl | 0.42-1.49 | 0.25-0.92 |
| BBS |  |  |
| Remaining merged SMD | 0.73 | 0.79 |
| P | 0.003 | 0.04 |
| 95%Cl | 0.24-1.22 | 0.03-1.54 |
| FMA-UE |  |  |
| Remaining merged SMD | 0.62 | 0.45 |
| P | 0.008 | 0.004 |
| 95%Cl | 0.16-1.07 | 0.14-0.76 |


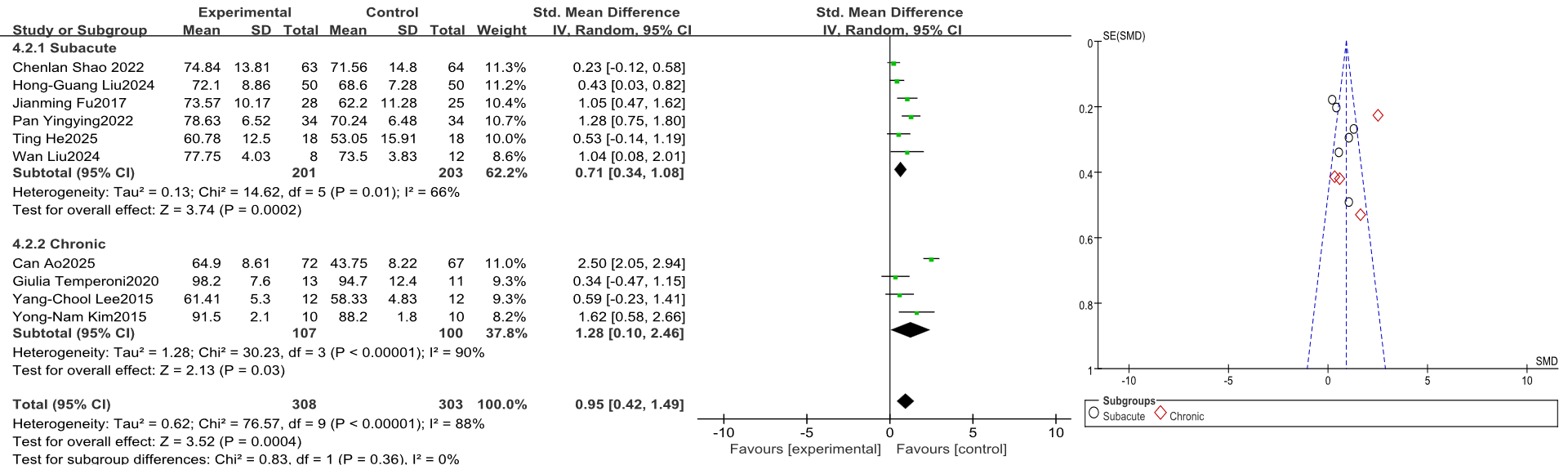


Figure S2 MBI Subgroup by Stroke Stage


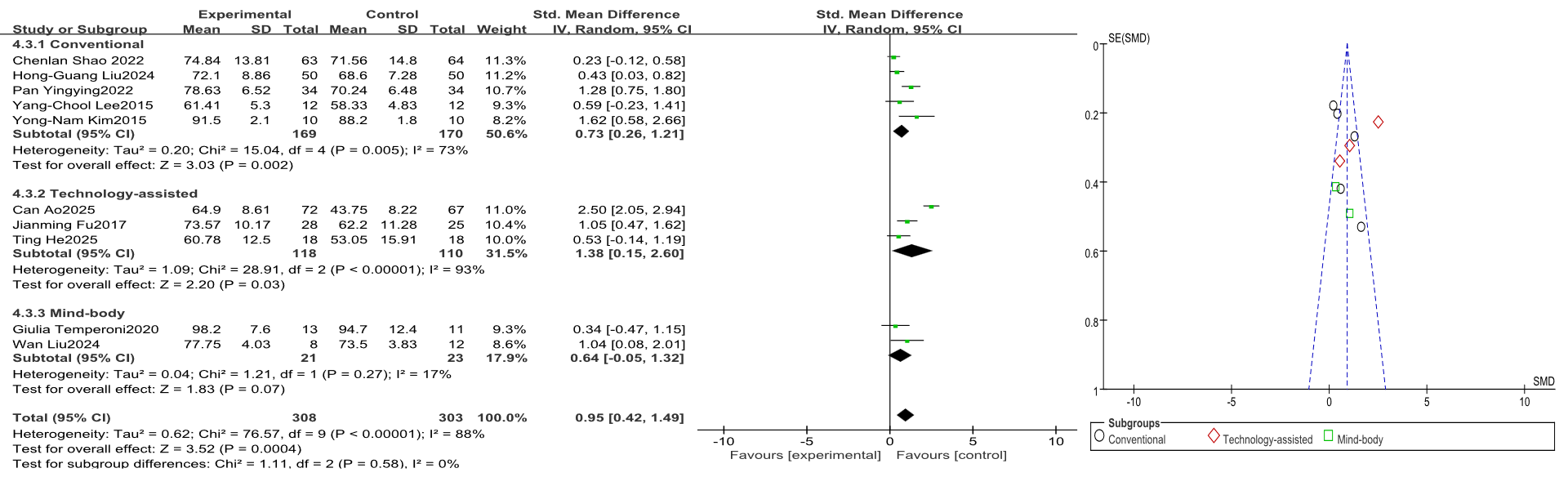


Figure S3 MBI Subgroup by Exercise Type


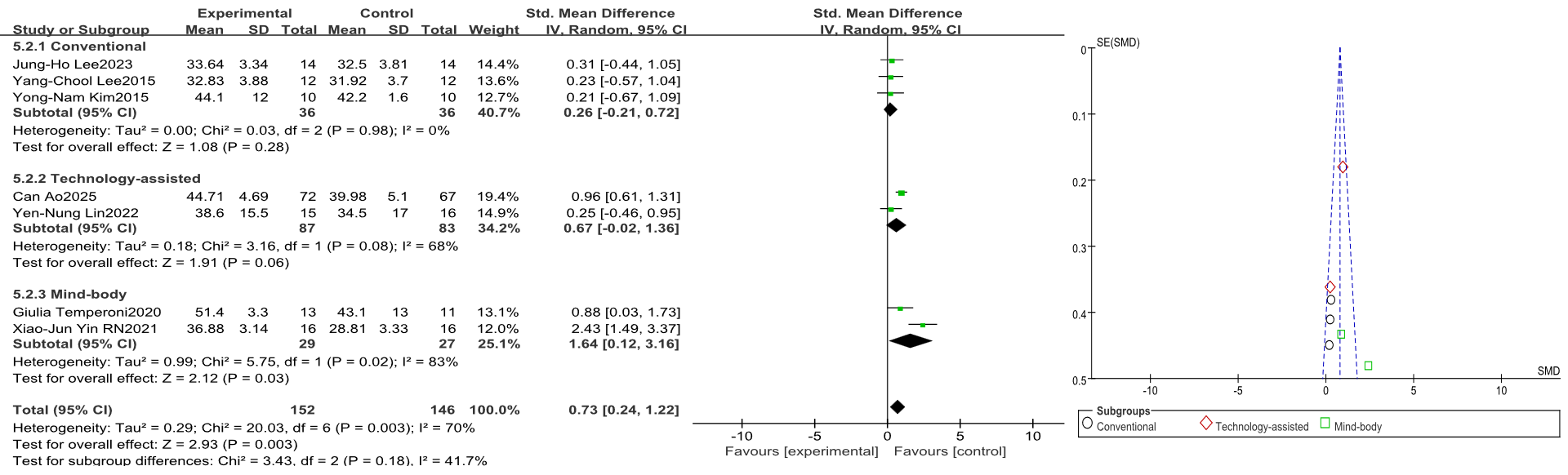


Figure S4 BBS Subgroup by Exercise Type


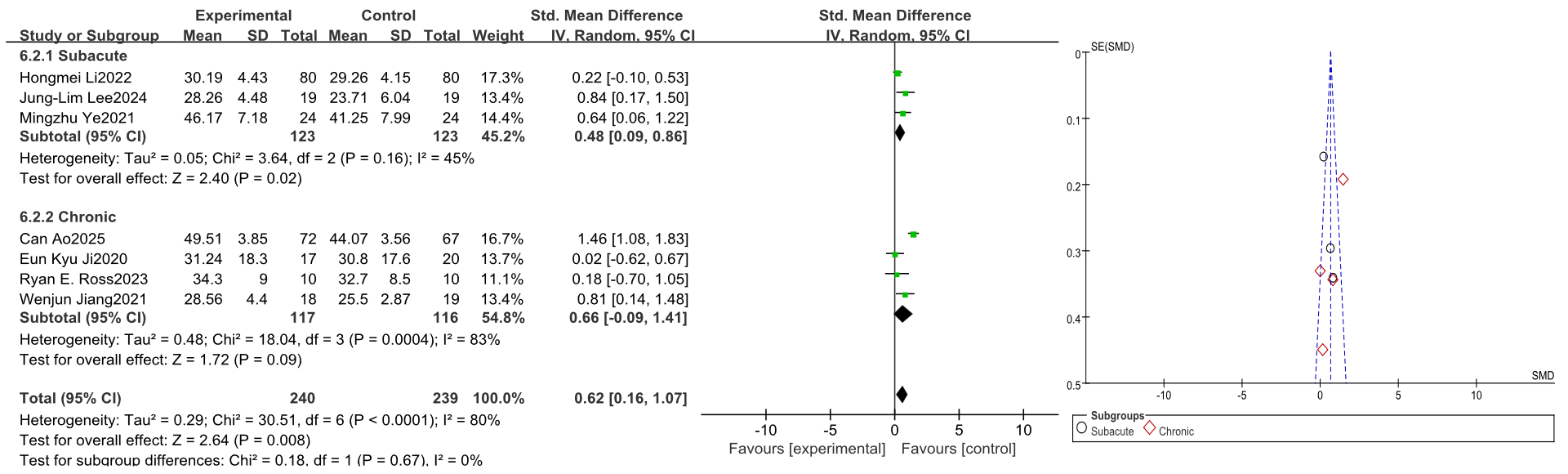


Figure S5 FMA-UE Subgroup by Stroke Stage


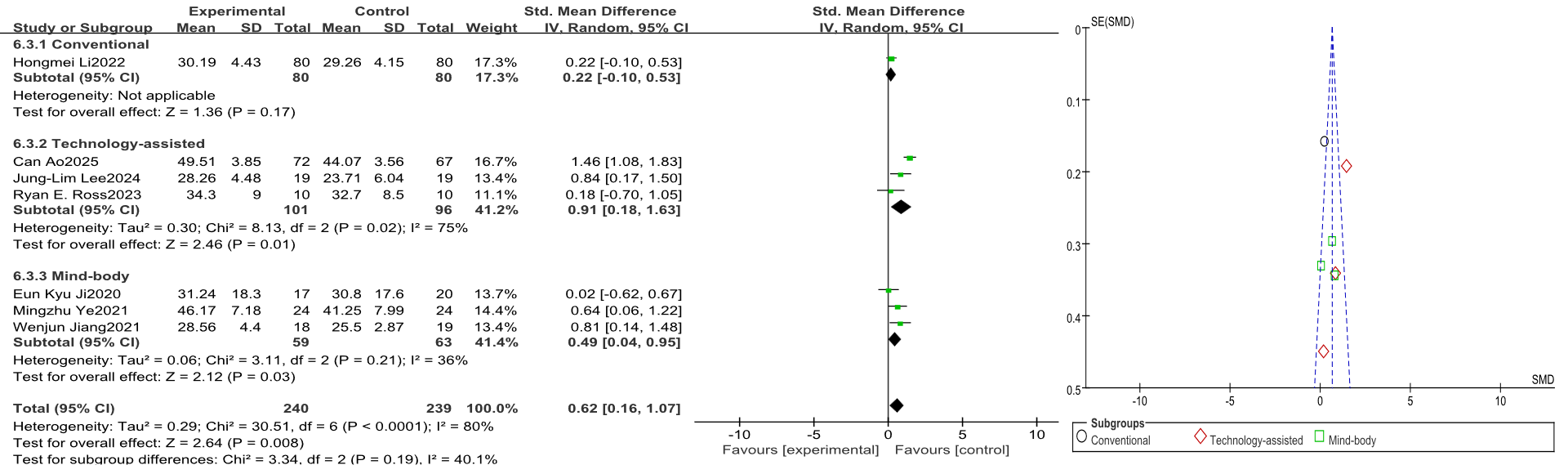


Figure S6 FMA-UE Subgroup by Exercise Type

Table S7 GRADE summary of findings

| Outcome | No. of studies | Study design | Risk of bias | Inconsistency | Indirectness | Imprecision | Publication bias | Certainty |
| --- | --- | --- | --- | --- | --- | --- | --- | --- |
| MBI | 10 | RCT | Serious (−1) | Serious (−1) | Not serious | Not serious | Serious (−1) | Very Low |
| BBS | 7 | RCT | Serious (−1) | Serious (−1) | Not serious | Serious (−1) | Serious (−1) | Very Low |
| FMA-UE | 7 | RCT | Serious (−1) | Serious (−1) | Not serious | Serious (−1) | Serious (−1) | Very Low |


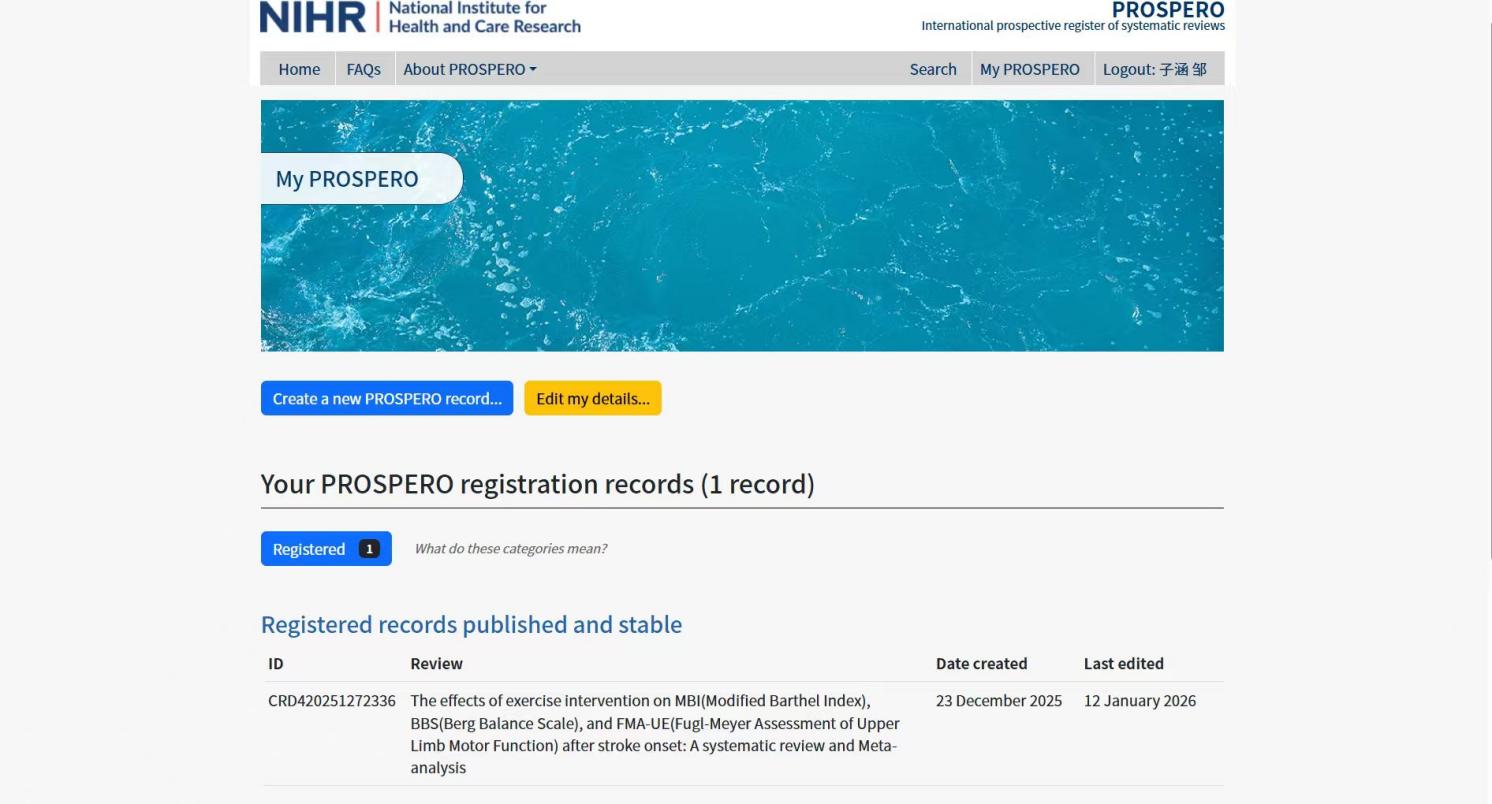


Figure 7 Search screenshot

Table S8 Full search strategies for each database.

| Database | Search Strategy |
| --- | --- |
| PubMed | ((“stroke”[MeSH Terms] OR “stroke”[Title/Abstract] OR “cerebrovascular accident”[Title/Abstract] OR “cerebral infarction”[Title/Abstract] OR “hemiplegia”[Title/Abstract] OR “hemiparesis”[Title/Abstract] OR “post-stroke”[Title/Abstract]) AND (“exercise”[MeSH Terms] OR “exercise therapy”[MeSH Terms] OR “physical therapy modalities”[MeSH Terms] OR “exercise”[Title/Abstract] OR “exercise therap”[Title/Abstract] OR “physical activity”[Title/Abstract] OR “aerobic training”[Title/Abstract] OR “resistance training”[Title/Abstract] OR “strength training”[Title/Abstract] OR “balance training”[Title/Abstract] OR “task-oriented training”[Title/Abstract] OR “robot-assisted”[Title/Abstract] OR “virtual reality”[Title/Abstract] OR “rehabilitation exercise”[Title/Abstract]) AND (“modified barthel index”[Title/Abstract] OR “Barthel index”[Title/Abstract] OR “MBI”[Title/Abstract] OR “berg balance scale”[Title/Abstract] OR “BBS”[Title/Abstract] OR “fugl-meyer assessment”[Title/Abstract] OR “FMA-UE”[Title/Abstract] OR “FMA”[Title/Abstract] OR “activities of daily living”[Title/Abstract] OR “upper limb function”[Title/Abstract] OR “motor function”[Title/Abstract] OR “motor recovery”[Title/Abstract])) AND (“randomized controlled trial”[Publication Type] OR “randomized”[Title/Abstract] OR “randomly”[Title/Abstract] OR “RCT”[Title/Abstract]). Filters: 2015/1/1 to 2025/3/31; English. |
| Web of Science | TS=(“stroke” OR “cerebrovascular accident” OR “cerebral infarction” OR “hemiplegia” OR “hemiparesis” OR “post-stroke”) AND TS=(“exercise” OR “exercise therap” OR “physical activity” OR “aerobic training” OR “resistance training” OR “strength training” OR “balance training” OR “task-oriented training” OR “robot-assisted” OR “virtual reality” OR “rehabilitation exercise”) AND TS=(“modified barthel index” OR “Barthel index” OR “MBI” OR “berg balance scale” OR “BBS” OR “fugl-meyer assessment” OR “FMA-UE” OR “FMA” OR “activities of daily living” OR “upper limb function” OR “motor function” OR “motor recovery”) AND TS=(“randomized controlled trial” OR “randomized” OR “randomly” OR “RCT”). Document types: Article. Timespan: 2015-01-01 to 2025-03-31. Languages: English. |
| Cochrane Library | #1 MeSH descriptor: [Stroke] explode all trees; #2 MeSH descriptor: [Exercise] explode all trees; #3 MeSH descriptor: [Exercise Therapy] explode all trees; #4 (stroke OR “cerebrovascular accident” OR “cerebral infarction” OR hemiplegia OR hemiparesis OR “post-stroke”):ti,ab,kw; #5 (exercise OR “exercise therap*” OR “physical activity” OR “aerobic training” OR “resistance training” OR “strength training” OR “balance training” OR “task-oriented training” OR “robot-assisted” OR “virtual reality”):ti,ab,kw; #6 (“modified barthel index” OR “Barthel index” OR “MBI” OR “berg balance scale” OR “BBS” OR “fugl-meyer assessment” OR “FMA-UE” OR “FMA” OR “activities of daily living” OR “upper limb function” OR “motor function”):ti,ab,kw; #7 (“randomized controlled trial” OR randomized OR randomly OR RCT):ti,ab,kw; #8 #1 OR #4; #9 #2 OR #3 OR #5; #10 #8 AND #9 AND #6 AND #7. Date limits: 2015–2025. |
| CNKI | SU=(‘卒中’+‘中风’+‘脑梗死’+‘脑血管意外’+‘偏瘫’)(‘运动’+‘运动训练’+‘运动疗法’+‘体育锻炼’+‘有氧训练’+‘抗阻训练’+‘平衡训练’+‘任务导向训练’+‘机器人辅助’+‘虚拟现实’+‘康复训练’)(‘改良Barthel指数’+‘MBI’+‘Berg平衡量表’+‘BBS’+‘Fugl-Meyer评估’+‘FMA-UE’+‘日常生活活动能力’+‘上肢功能’+‘运动功能’)*(‘随机对照’+‘RCT’+‘随机’). 时间限定：2015年1月1日至2025年3月31日. |

Abbreviations: CNKI, China National Knowledge Infrastructure; FMA-UE, Fugl-Meyer Assessment of Upper Extremity; MBI, Modified Barthel Index; BBS, Berg Balance Scale; RCT, randomized controlled trial; MeSH, Medical Subject Headings; TI, Title; AB, Abstract; KW, Keywords; TS, Topic; SU, Subject.
